# MYO7A is required for the functional integrity of the mechanoelectrical transduction complex in hair cells of the adult cochlea

**DOI:** 10.1073/pnas.2414707122

**Published:** 2025-01-02

**Authors:** Anna Underhill, Samuel Webb, Fiorella C. Grandi, Jing-Yi Jeng, Jacques B. de Monvel, Baptiste Plion, Adam J. Carlton, Ana E. Amariutei, Niovi Voulgari, Francesca De Faveri, Federico Ceriani, Mirna Mustapha, Stuart L. Johnson, Saaid Safieddine, Corné J. Kros, Walter Marcotti

**Affiliations:** ^a^School of Biosciences, University of Sheffield, Sheffield S10 2TN, United Kingdom; ^b^Sorbonne Université, INSERM, Institute de Myologie, Centre de Researche en Myologie, Paris F-75013, France; ^c^Université de la Cité de Paris, Institut Pasteur, Assistance publique - Hôpitaux de Paris, Inserm, Fondation pour l’audition, CNRS, Instituts Hospitalo-Universitaires reConnect, Paris F-75012, France; ^d^Neuroscience Institute, University of Sheffield, Sheffield S10 2TN, United Kingdom; ^e^School of Life Sciences, University of Sussex, Falmer, Brighton BN1 9QG, United Kingdom

**Keywords:** cochlea, hair cell, stereocilia, mechanoelectrical transduction, hearing loss

## Abstract

Myosin-VIIA (MYO7A) is an unconventional myosin responsible for syndromic (Usher 1B) or nonsyndromic forms of deafness in humans when mutated. In the cochlea, MYO7A is expressed in hair cells, where it is believed to act as the motor protein tensioning the mechanoelectrical transducer (MET) channels, thus setting their resting open probability (*P_o_*). However, direct evidence for this unique role for an unconventional myosin in mature hair cells is lacking. Here, we show that MYO7A has a distinct role in hair cells, being crucial for the structural integrity of hair bundles. Postnatal deletion of *Myo7a* leads to 87 to 96% reduction in MYO7A from hair cells by postnatal day 20 (P20), without affecting hearing function. During the following week, mice showed progressive decline in both hearing function and MET current amplitude in hair cells without affecting the resting *P_o_* and calcium sensitivity of the MET channel. Hair-bundle stiffness was normal at P20 but halved at P30, despite it having a normal staircase morphology and tip links. The reduction of MYO7A in the stereocilia (>87%) increased their vulnerability to sound-induced damage, with significantly more hearing loss and hair bundle deterioration than in control mice. RNA-sequencing identified a downregulation of several stereociliary genes in the *Myo7a*-deficient cochlea, indicating the presence of indirect compensatory mechanisms. This study reveals that mature hair cells seem to use a MYO7A-independent mechanism to maintain the resting *P_o_* of the MET channels. Instead, MYO7A is essential for maintaining the structural and functional integrity of the hair bundles.

## Significance

In hair cells, the gating of the mechanoelectrical transducer (MET) channels requires force supplied by the tensioning of tip links during sound-induced displacement of the hair-cell stereociliary bundles. The motor protein MYO7A has long been associated with tip-link tensioning, but conclusive evidence is lacking. Here, we investigated the role of MYO7A in mature hair cells using conditional knockout mice. The reduced level (>87%) of MYO7A caused the progressive loss of the MET current without affecting the fraction of the current active when the bundle is in its resting position. Loss of MYO7A also resulted in increased vulnerability of hair cells to sound-induced damage. We conclude that in hair cells MYO7A is crucial for the structural integrity of the MET complex.

The transduction of acoustic information into electrical signals depends on the mechanically induced displacement of stereociliary bundles projecting from the apical surface of the sensory hair cells (1). Hair bundle deflection opens mechanoelectrical transducer (MET) channels located at the tips of the shorter rows of adjacent stereocilia (2). Stereocilia are specialized microvilli-like structures with a cytoskeletal core composed of cross-linked and uniformly polarized actin filaments (3, 4). In the mouse cochlea, the characteristic staircase-like structure of the hair bundle usually includes three rows of stereocilia interconnected by several extracellular linkages (3, 5). One of these extracellular filaments is the tip link, which is formed by cadherin 23 (CDH23) and protocadherin 15 (PCDH15) at the upper and lower part, respectively (6–8). The tip links transmit force from the mechanical displacement of the stereociliary bundles to the MET channel. Current evidence indicates that TMC1/2 and TMIE contribute to the pore-forming subunits of the MET channel, although the MET channel complex harbors several other proteins essential for mechanoelectrical transduction (1). At the other end of the tip link, CDH23 interacts with the upper tip-link complex located on the side of the taller stereocilia. This complex is composed of the F-actin-bound myosin motor MYO7A and the adaptor proteins USH1C (Harmonin) and USH1G (Sans), which provide the scaffolding required for MYO7A to interact with the tip links (9–11).

MYO7A is an unconventional myosin expressed in both the inner hair cells (IHCs) and outer hair cells (OHCs) of the mammalian cochlea (refs. 12–14, gEAR: https://umgear.org). However, recent evidence indicates that IHCs and OHCs express different isoforms of MYO7A (15). Mutations in *Myo7a* cause syndromic (Usher 1B) or nonsyndromic deafness in humans (16–18) and deafness in mice (19). Constitutive Usher1 mouse mutants, including those for *Myo7a*, are profoundly deaf and their hair cells show early and severe morphological defects in the hair bundles (20). Apart from the key role of these Usher proteins in hair bundle development, single-cell functional studies have implied that both MYO7A and Harmonin are required for setting the resting open probability of the MET channels (9, 15, 21). These findings led to the conclusion that the upper tip link complex acts as the force generator that, by tensioning the tip links, keeps the open probability of the MET channel within the most sensitive part of the current–displacement relationship. However, the role of MYO7A in mechanoelectrical transduction in adult mice, without the confounding effects of abnormal morphological hair cell bundle development (21, 22) or residual expression of MYO7A (15, 23), is still not well understood.

In this study, we investigated the role of MYO7A in mature hair cells from conditional knockout mice in which the gradual decline in the amount of the protein allowed normal cochlear development and hearing function up to about postnatal day 20 (P20). By P20, however, which is about 17 d following cre-dependent recombination driven by the *Myo15* promoter (11), MYO7A was already reduced in the hair cells by >87%. In the following few days, hair cells from *Myo7a*-deficient mice progressively lose their MET current despite having normal hair bundle morphology, including the presence of tip links, up to at least 1 mo of age, albeit with a considerably reduced stiffness. Surprisingly, the resting open probability of the MET channel and its sensitivity to intracellular and extracellular Ca^2+^ were not affected by the >87% reduction in MYO7A. By 2 mo of age, the hair bundles of the hair cells started to become disorganized and by 7 mo the organ of Corti was almost completely devoid of hair cells. We also found that noise insults accelerated the progression of hearing loss and deterioration of the stereociliary hair bundles in *Myo7a*-deficient mice. Transcriptomic analysis in 1-mo-old *Myo7a*-deficient mice highlighted the downregulation of genes known to be essential for mechanoelectrical transduction. We propose that MYO7A is required for maintaining the functional integrity of the stereociliary hair bundles.

## Results

### Progressive Loss of Cochlear Function in *Myo7a*-Deficient Mice.

**Fig. 1.**
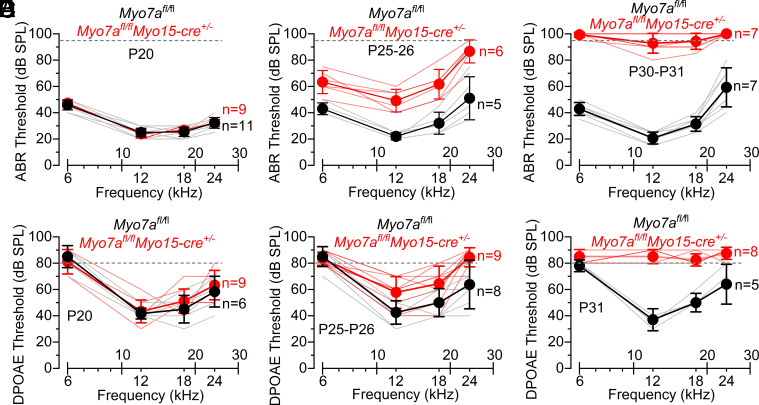
Hearing thresholds become progressively elevated in *Myo7a*-deficient mice. (*A–C*) ABR thresholds for frequency-specific pure tone burst stimuli recorded from controls *Myo7a^fl/fl^* and *Myo7a^fl/fl^Myo15-cre^+/−^* mice at P20 (*A*), P25 to P26 (*B*), and P30 to P31 (*C*). (*D–F*) DPOAE thresholds measured from P20 (*A*), P25 to P26 (*B*), and P31 (*C*) *Myo7a^fl/fl^* controls and *Myo7a^fl/fl^Myo15-cre^+/−^* mice. The frequencies tested for both ABRs and DPOAEs were 6, 12, 18, and 24 kHz. The dashed line represents the upper threshold limit: 95 dB and 80 dB for ABRs and DPOAEs, respectively. The number of mice tested for each genotype is shown next to the data. Data are plotted as mean ± SD.

To investigate the role of MYO7A in adult cochlear hair cells, we used a targeted knockout obtained by crossing *Myo7a* floxed mice (*Myo7a^fl/fl^*) with *Myo15-cre^+/−^* mice (11, 23). In these mice, cre-dependent recombination, which is driven by the hair cell–specific *Myo15* promoter, occurs from about postnatal day 3 to 4 (P3 to P4) in the apical coil of the cochlea (11). Despite the progressive decline in the amount of MYO7A in the hair cells, the stereociliary bundle developed normally and acquired a staircase profile indistinguishable from that of control mice at least up to just over 1 mo of age (23). However, by 2 mo of age, 36% of the OHC stereociliary hair bundles, but not those of IHCs, were absent in *Myo7a^fl/fl^Myo15-cre^+/−^* mice (*SI Appendix,* Fig. S1 *A* and *B*). At 6 mo of age, only very few disorganized hair bundles remained in both OHCs and IHCs of *Myo7a^fl/fl^Myo15-cre^+/−^* mice (*SI Appendix,* Fig. S1 *C* and *D*), which resembles the phenotype observed in the neonatal cochlea from constitutive *Myo7a* knockout mice (21, 22). We then investigated the time course of hearing loss of *Myo7a^fl/fl^Myo15-cre^+/−^* mice using auditory brainstem responses (ABRs) and distortion product otoacoustic emissions (DPOAEs). Thresholds for pure-tone evoked ABRs were similar between *Myo7a^fl/fl^* and *Myo7a^fl/fl^Myo15-cre^+/−^* mice at P20 (*P* = 0.6467, two-way ANOVA, Fig. 1*A*), but significantly elevated in the latter at both P25 and P30 (*P* < 0.0001 for both comparisons, Fig. 1 *B* and *C* and *SI Appendix,* Fig. S2). Similarly, DPOAE thresholds were not significantly different between the two genotypes at P20 (*P* = 0.3764, Fig. 1*D*), but were greatly elevated by P25 to P26 (*P* < 0.0001, Fig. 1*E*), with responses being almost completely absent at P31 (*P* < 0.0001, Fig. 1*F* and *SI Appendix,* Fig. S3). Both *Myo7a^fl/fl^* and *Myo15-cre^+/−^* mice exhibit normal hearing function and their hair cells have normal biophysical properties (23). These results indicate that the hair cells are likely to function normally up to around P20.

### Progressive Decrease of MYO7A in the Hair Cells of *Myo7a*-Deficient Mice.

Recording MET currents in mature hair cells requires the removal of the tectorial membrane (TM), which is a strip of extracellular matrix that lies on top of the sensory epithelia preventing the displacement of the hair bundles. Moreover, the TM attaches to the tips of the tallest row of OHC stereocilia from just before the onset of hearing, causing damage to the hair bundles upon its physical removal. Therefore, MET recordings were primarily done using mice in which TECTA, a major noncollagenous component of the TM, had been knocked out (*Tecta^−/−^*) (24). *Tecta^−/−^* mice still have a TM, but it is detached from the cochlear sensory epithelium and the OHC stereocilia (24, 25). In mice with a detached TM, the staircase structure of the hair bundles of mature OHCs was preserved and the MET current retains normal resting open probability and Ca^+^ sensitivity (25). Here, we provide further evidence that the size and resting open probability of the MET current in the OHCs were indistinguishable between control mice (*Myo7a^fl/fl^*) and those with a detached TM (*Myo7a^fl/fl^Tecta^−/−^*) (*SI Appendix,* Fig. S4).

**Fig. 2.**
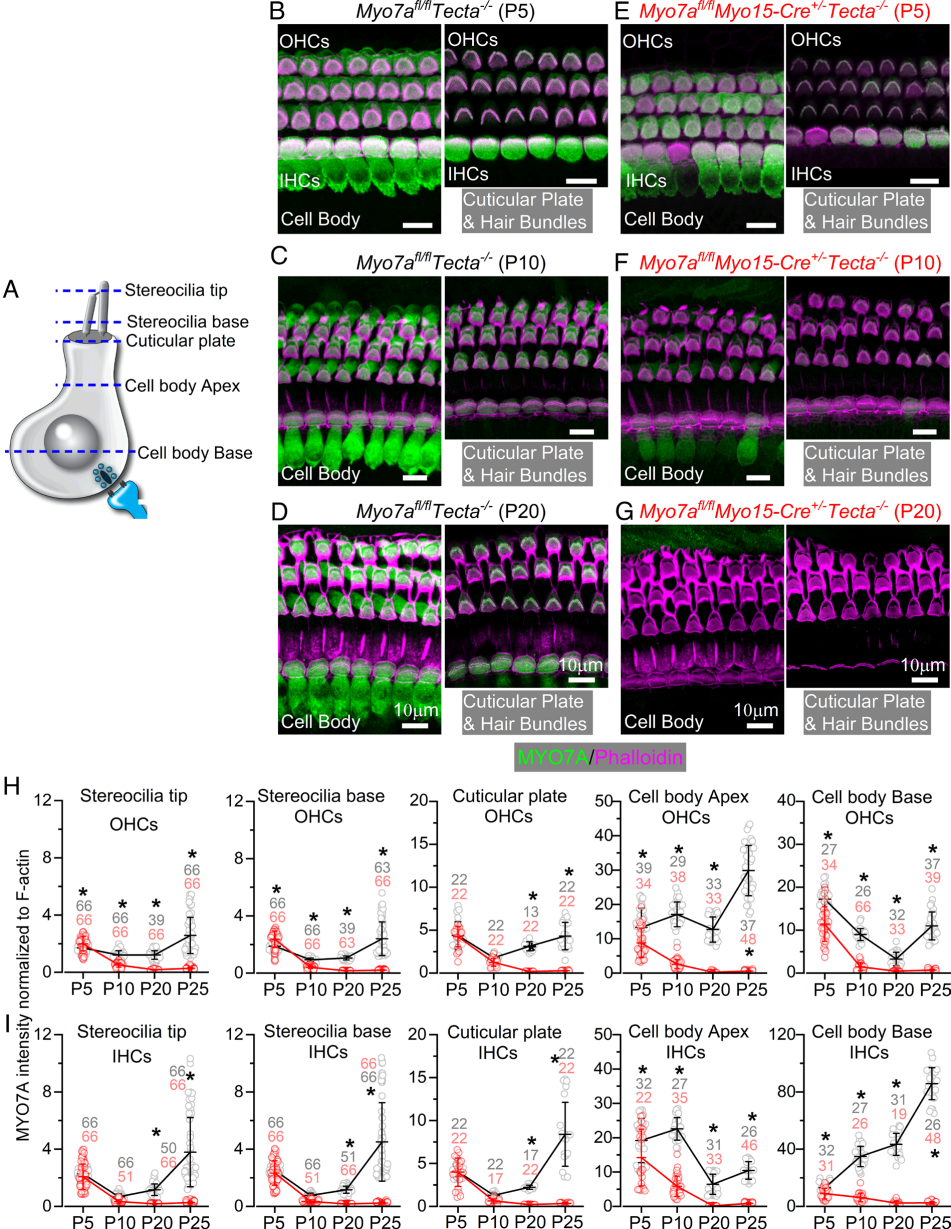
Time course of downregulation of MYO7A in *Myo7a^fl/fl^Tecta^−/−^* mice (*A*) Schematic representation of a hair cell showing the five positions along the confocal z-stack used to quantify MYO7A fluorescence intensity: i) along stereocilia: stereocilia bases and stereocilia tips; for the latter, images were taken toward the tips of the longest stereocilia, but above those of the middle row; ii) cuticular plate; iii) apical and basal cell body regions. (*B–G*) Confocal images showing the MYO7A immunostaining (green) and F-actin (phalloidin, magenta) in both OHCs and IHCs from control (*Myo7a^fl/fl^Tecta^−/−^*: *B–D*) and knockout mice (*Myo7a^fl/fl^Myo15-cre^+/−^Tecta^−/−^*: *E–G*) at P5 (*B* and *E*), P10 (*C* and *F*), and P20 (*D* and *G*). For each panel, images show MYO7A immunostaining at the cell body (*Left*) and both cuticular plate and hair bundles (*Right*). (*H* and *I*) Average MYO7A intensity normalized to F-actin taken at different ages (P5, P10, P20, and P25), cellular positions (as in panel *A*) along the OHCs (*H*) and IHCs (*I*) from control (black) and *Myo7a^fl/fl^Myo15-cre^+/−^Tecta^−/−^* (red) mice. Each data-point represents a local average of MYO7A intensity computed in a small Gaussian volume centered at a given position, which was divided by the corresponding F-actin intensity averaged in the same Gaussian volume (SD along the x-y and z positions: 250 nm and 500 nm for stereocilia positions; 1 µm and 500 nm for the cuticular plate; 1 µm and 1 µm for cytoplasmic positions). Error bars represent one SD around the mean computed for each stage. *Indicate significant difference from the Šídák’s post test (two-way ANOVA): *P* < 0.0001 for all ages in OHCs and IHCs apart for cuticular plate P20 IHCs (*P* = 0.0002) and cytoplasm base P5 IHCs (*P* = 0.0171). Number of hair bundle/hair cell tested is shown above or below the data (at least three mice per genotype and age).

The time course of MYO7A reduction in mice with a detached TM (*Myo7a^fl/fl^Myo15-cre^+/−^Tecta^−/−^*) was investigated by immunostaining the hair cells (Fig. 2 *A*–*I*) with two MYO7A antibodies (*SI Appendix*, *Methods*). We determined the fluorescence intensity of MYO7A at five different horizontal planes along the hair cell length (Fig. 2 *A*–*G*), some of which have previously been linked to a specific role for MYO7A (10, 26, 27). At P5, MYO7A immunostaining showed comparable level in most hair cell regions between control (*Myo7a^fl/lf^Tecta^−/−^*) and *Myo7a^fl/fl^Myo15-cre^+/−^Tecta^−/−^* mice (Fig. 2 *B*, *E*, *H*, and *I*). The expression level of MYO7A was significantly decreased in most hair cell regions at P10 (Fig. 2 *C*, *F*, *H*, and *I*). By P20, which is a time when the ABR and DPOAEs are still indistinguishable between the two genotypes (Fig. 1), MYO7A was almost undetectable throughout the IHCs and OHCs of *Myo7a^fl/fl^Myo15-cre^+/−^Tecta^−/−^* mice (Fig. 2 *D*–*I*). On average, MYO7A immunostaining was reduced between 87% and 96% at P20 in the hair cells of *Myo7a^fl/fl^Myo15-cre^+/−^Tecta^−/−^* mice compared to littermate controls and remained at comparable values in P25 mice (88 to 98%). Qualitatively similar results were also seen in *Myo7a^fl/fl^Myo15-cre^+/−^* (*SI Appendix,* Fig. S5) (23).

### Reduced Size and Normal Resting MET Current in Mature OHCs from Mice Lacking MYO7A.

**Fig. 3.**
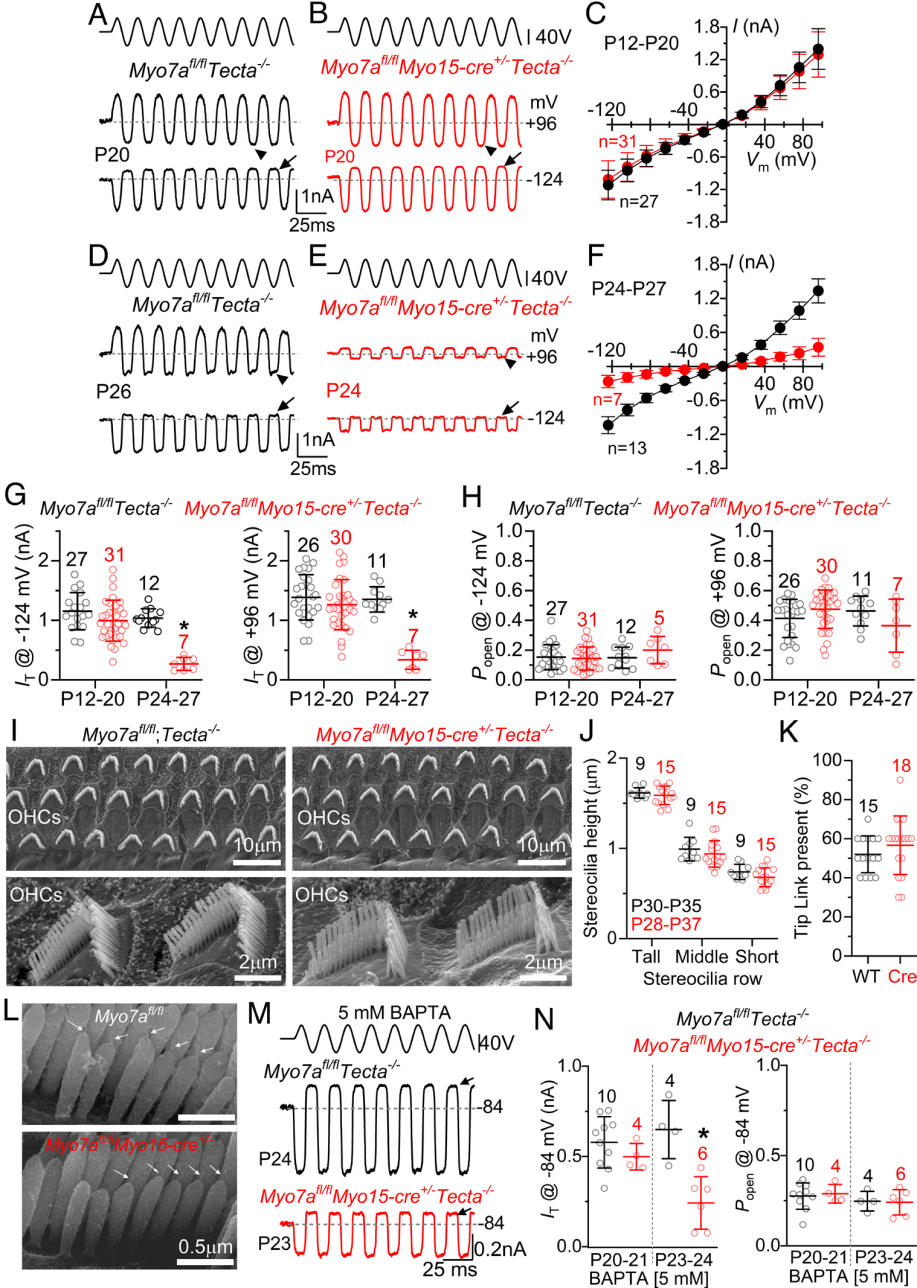
Mechanoelectrical transduction in OHCs from adult *Myo7a* knockout mice. (*A* and *B*) Saturating MET currents recorded from OHCs of P20 control (*A*: *Myo7a^fl/fl^Tecta^−/−^*) and knockout mice (*B*, *Myo7a^fl/fl^Myo15-cre^+/−^Tecta^−/−^*). MET currents were elicited using sinusoidal force stimuli to the hair bundles at membrane potentials between −124 mV and +96 mV (20 mV increments from −84 mV and with 1 mM intracellular EGTA). Driver voltage (DV) stimuli to the fluid jet are shown above the traces (positive DV being excitatory). Arrows and arrowheads indicate the closure of the MET channel during inhibitory stimuli at −124 mV and +96 mV, respectively. (*C*) Average peak-to-peak MET current-voltage curves, obtained as described in panels *A* and *B*, from control (27 OHCs, 22 mice) and *Myo7a^fl/fl^Myo15-cre^+/−^Tecta^−/−^* (31 OHCs, 22 mice) OHCs. (*D–F*) Saturating MET currents recorded from OHCs as described in panels *A–C,* but from P26 control (*D* and *F*, 12 OHCs, 11 mice) and P24 *Myo7a^fl/fl^Myo15-cre^+/−^Tecta^−/−^* mice (*E* and *F*, seven OHCs, six mice). (*G*) Maximal MET current size in the OHCs from both genotypes tested at −124 mV (*Left*) and +96 mV (*Right*). Genotype comparison at −124 mV: P12 to P20, *P* = 0.1849, and P24 to P27, *P* < 0.0001; at +96 mV: P12 to P20, *P* = 0.3734, and P24 to P27 *P* < 0.0001 (Šídák’s post test, two-way ANOVA). (*H*) Resting MET current open probability (*P_open_*) in OHCs from both genotypes, which is the fraction present in the absence of mechanical stimulation (measured as the holding current minus the current present during inhibitory bundle deflection divided by the maximal MET current). Genotype comparison at −124 mV (*Left*): P12 to P20, *P* = 0.8891, and P24 to P27 *P* = 0.3298; at +96 mV (*Right*): P12 to P20. *P* = 0.1459 and P24 to P27, *P* = 0.2260). (*I*) SEM images showing the OHC hair bundle from P28 to P37 *Myo7a^fl/fl^Tecta^−/^* and *Myo7a^fl/fl^Myo15-cre^+/−^Tecta^−/−^* (*Bottom* panels: higher magnification images). (*J*) Height of the three rows of stereocilia in the OHCs of P28 to P37 mice from both genotypes (control: 9 bundles/OHCs from, 3 cochleae, 3 mice; *Myo7a^fl/fl^Myo15-cre^+/−^Tecta^−/−^*: 15 bundles/OHCs from, 5 cochleae, 5 mice). Single data points: individual bundle measurements. (*K* and *L*) Percentage of tip links measured between 10 pairs of adjacent stereocilia per bundle in the 2nd and 3rd rows (shortest two rows) (*K*) and SEM images showing the presence of the tip links (*L*) from P28 to P37 control (*Myo7a^fl/fl^* and *Myo7a^fl/fl^Tecta^−/−^*: 15 OHCs, 5 mice) and knockout mice (*Myo7a^fl/fl^Myo15-cre^+/−^* and *Myo7a^fl/fl^Myo15-cre^+/−^Tecta^−/−^*: 18 OHCs, 6 mice); the percentages were not significantly different (*K*: *P* = 0.2278, Mann–Whitney *U* test). The number of tip links between the 1st and 2nd rows of stereocilia was also not significantly different (*P* = 0.5492) between control (25 ± 12%, 16 OHCs, 5 mice) and *Myo7a*-deficient mice (28 ± 9%, 12 OHCs, 5 mice). (*M*) MET currents recorded from OHCs (as described in panels *A* and *B*) from both genotypes at −84 mV in the presence of 5 mM intracellular BAPTA. Note the large resting MET current (arrows). (*N*) Maximal size of the MET current (*Left*) and *P_open_* (*Right*) recorded from OHCs of both genotypes. Data in panels *C–H*, *J*, *K*, and *N* are plotted as mean ± SD.

Using the above *Myo7a^fl/fl^Tecta^−/−^* mouse strain, we investigated the functional changes in the MET current of OHCs from posthearing mice due to the knockout of *Myo7a* (Fig. 3 *A*–*H*). The MET current was recorded in the presence of 1 mM intracellular EGTA and elicited by displacing the OHC hair bundles using a 50 Hz sinusoidal force stimulus from a piezo-driven fluid jet (28). At negative membrane potentials, moving the bundles toward the taller stereocilia (i.e., in the excitatory direction) elicited a large inward MET current in OHCs of both *Myo7a^fl/fl^Tecta^−/−^* and *Myo7a^fl/fl^Myo15-cre^+/−^Tecta^−/−^* P20 mice (Fig. 3 *A*–*C*). By stepping the membrane potential from −124 mV to more depolarized values in 20 mV increments, the MET current decreased in size at first and then reversed near 0 mV in both genotypes (Fig. 3*C*), consistent with the nonselective permeability of MET channels to cations. For voltage steps positive to 0 mV, the MET current became outward during excitatory bundle stimulation (Fig. 3 *A*–*C*). The size of the MET current in P12 to P20 OHCs was not significantly different between control *Myo7a^fl/fl^Tecta^−/−^* and *Myo7a^fl/fl^Myo15-cre^+/−^Tecta^−/−^* mice (Fig. 3 *C* and *G*). This result is consistent with the normal ABR and DPOAE thresholds in P20 *Myo7a*-deficient mice (Fig. 1) despite the large reduction (>87%) in MYO7A along the stereociliary bundles (Fig. 2). A few days later (P24 to P27), the size of the MET current in the OHCs from *Myo7a^fl/fl^Myo15-cre^+/−^Tecta^−/−^* mice was much reduced compared to controls (*Myo7a^fl/fl^Tecta^−/−^*: Fig. 3 *D*–*F*), which explains the decreased hearing of *Myo7a*-deficient mice at P25 to P26 (Fig. 1). However, the proportion of MET current active at rest (i.e., the resting open probability, *P_open_*) was not significantly affected at both negative and positive membrane potentials (Fig. 3*H*).

The loss of the MET current in P24 to P27 OHCs was not due to morphological defects of the stereociliary bundles lacking MYO7A since their height was not significantly different between control and *Myo7a*-deficient P28 to P37 mice (*P* = 0.0896, two-way ANOVA, Fig. 3 *I* and *J*). The width of the hair bundles was also not significantly different for all three rows of OHCs between the two genotypes (*SI Appendix,* Fig. S6*A*). We also assessed the number of tip links in the OHC hair bundle by SEM from P28 to P37 control (*Myo7a^fl/fl^* and *Myo7a^fl/fl^Tecta^−/−^*) and knockout mice (*Myo7a^fl/fl^Myo15-cre^+/−^* and *Myo7a^fl/fl^Myo15-cre^+/−^Tecta^−/−^*). We found that the percentage of tip links present in the bundle (*SI Appendix*, *Methods*) was not significantly different between control and knockout mice (Fig. 3 *K* and *L**, P* = 0.2278, Mann–Whitney *U* test).

Calcium entry via the resting MET current induces a degree of adaptation, which leads to the partial closure of the MET channels and a reduction of resting *P_open_* (29). A manifestation of this phenomenon is the increased resting *P_open_* of the MET channel at positive membrane potentials, which is when Ca^2+^ influx into the stereocilia is decreased because the cell is closer to the reversal potential for Ca^2+^. To investigate whether Ca^2+^-dependent adaptation was affected in adult OHCs lacking MYO7A, we directly interfered with the level of extracellular Ca^2+^ or free Ca^2+^ in the stereociliary bundle by changing the intracellular Ca^2+^ buffering capacity with the fast Ca^2+^ buffer BAPTA. We found that the MET current in 5 mM intracellular BAPTA was significantly smaller in OHCs from P23 to P24 *Myo7a^fl/fl^Myo15-cre^+/−^Tecta^−/−^* compared to control *Myo7a^fl/fl^Tecta^−/−^* mice (*P* = 0.0004, Šídák’ post test, two-way ANOVA, Fig. 3 *M* and *N*). However, the large resting *P_open_* of the MET current in 5 mM BAPTA was not significantly different between the two ages and genotypes (*P* = 0.9063, two-way ANOVA, Fig. 3 *M* and *N*), demonstrating that a reduced Ca^2+^ entry into the MET channel significantly increases its resting open probability. This further supports the finding that MYO7A does not contribute to setting the resting *P_open_* of the MET current in adult OHCs.

A functional MET channel has been shown to be essential for maintaining the basolateral membrane biophysical characteristics of adult IHCs from *Myo7a*-deficient mice (23). Here, we found that the mature OHCs also require a functional MET current (*SI Appendix,* Fig. S7).

### Hair Bundle Stiffness Is Reduced in OHCs from *Myo7a* Knockout Mice.

**Fig. 4.**
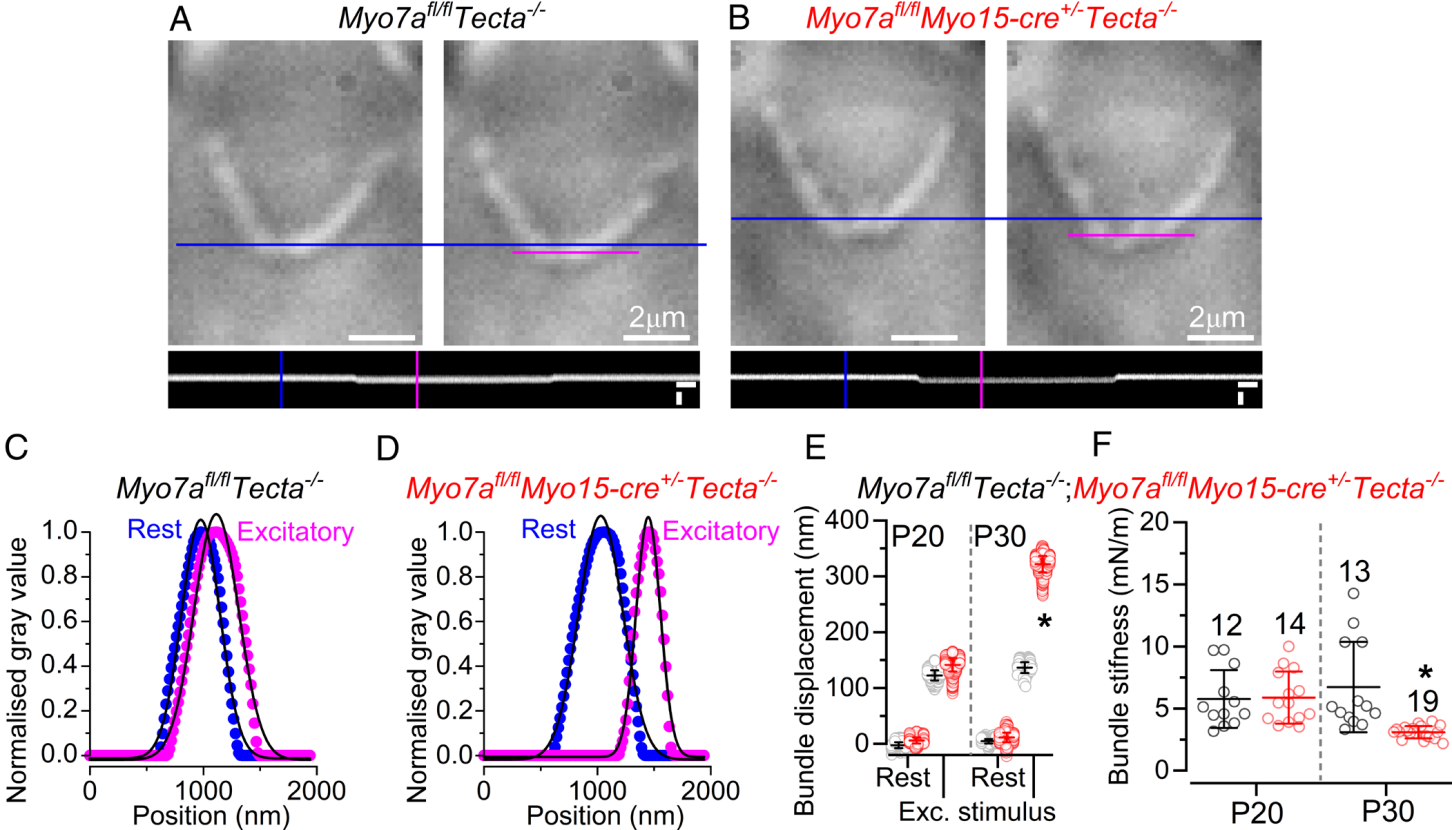
Adult OHCs from *Myo7a* knockout mice have reduced hair bundle stiffness. (*A* and *B*) Images of the OHCs hair bundle position at rest (blue lines), and during saturating excitatory stimuli (magenta lines) using a fluid jet from control (*A*) and *Myo7a^fl/fl^Myo15-cre^+/–^Tecta^–/–^* mice (*B*). Bottom horizontal panels represent the z to y (time-series displacement) reslices of *A* (control) and *B* (*Myo7a^fl/fl^Myo15-cre^+/−^Tecta^−/−^*). Scale bars: 400 nm (*y* axis), 1 ms (*x* axis). (*C* and *D*) Individual frame examples taken from the blue and magenta lines in *A* and *B* (horizontal panels) and plotted as normalized gray values against position. Bundle position was determined by fitting the data with a Gaussian amplitude function. (*E*) Bundle displacement obtained by plotting 900 frames from both the resting and excitatory position from one OHC per genotype (from *A* and *B*) using the process from *C* and *D.* (*F*) Stiffness of each individual OHC hair bundle (*SI Appendix*, *Methods*), which was significantly reduced in P30 *Myo7a^fl/fl^Myo15-cre^+/−^Tecta^−/−^* mice. Number of OHCs shown above the data points apply to panels *F* (P20: 4 mice/genotype; P30: 5 mice/genotype).

Considering that the reduced level (>87%) of MYO7A from the OHCs does not affect the morphology of their stereociliary bundles, at least up to about P37 (Fig. 3 *I* and *J*), we investigated the mechanical properties of the hair bundles in P20 and P30 mice (Fig. 4 and *SI Appendix,* Fig. S8). For these experiments, OHC hair bundles were displaced using force steps delivered by the fluid-jet used for MET recordings, and their movement was recorded using a fast camera (*Materials and Methods*). We found that at P30, but not at P20, the same fluid jet step stimuli elicited much larger bundle movements in OHCs from *Myo7a^fl/fl^Myo15-cre^+/−^Tecta^−/−^* mice compared to those in *Myo7a^fl/fl^Tecta^−/−^* controls (Fig. 4 *A*–*E* and *SI Appendix,* Fig. S8). The apparent overall steady-state bundle stiffness of the OHCs (see *Materials and Methods* for details), which was measured at the end of the step-displacement stimulus, was comparable between the two genotypes at P20 (*P* = 0.9900, Šídák’ post test, two-way ANOVA, Fig. 4*F*), but significantly reduced at P30 compared to controls (*P* < 0.0001, Fig. 4*F*). Since tip links are still present in *Myo7a*-deficient mice, the decreased hair bundle stiffness at P30, but not at P20, is likely to be a consequence of some unknown morphological changes in the bundle structure.

### Mature IHCs from *Myo7a^fl/fl^Myo15-cre^+/−^Tecta^−/−^* Also Exhibit Reduced Size and Normal Resting *P_open_* of the MET Current.

**Fig. 5.**
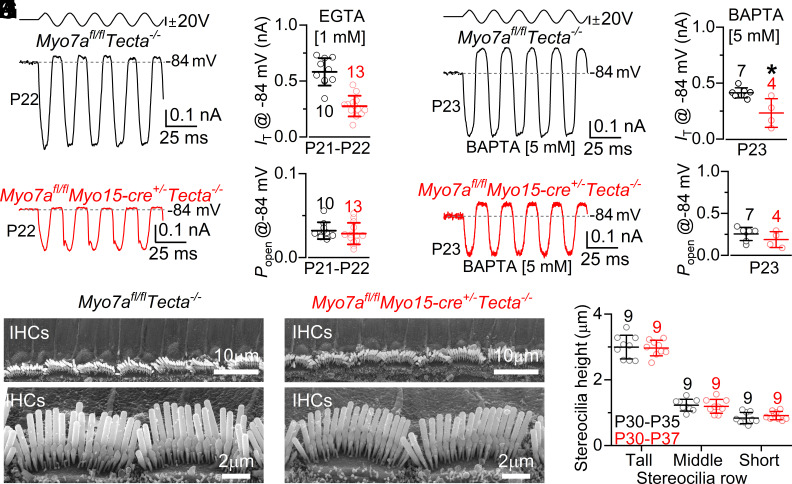
Mechanoelectrical transduction in IHCs from adult *Myo7a* knockout mice. (*A* and *B*) Saturating MET currents recorded from IHCs of P22 control (*A*) and *Myo7a^fl/fl^Myo15-cre^+/−^Tecta^−/−^* mice (*B*) at −84 mV and with 1 mM intracellular EGTA. (*C* and *D*) Size of the MET current (*C*) and resting *P*_open_ (*D*) recorded from IHCs from P21 to P22 mice of both genotypes. (*E* and *F*) MET currents recorded from IHCs of a control (*E*) and a *Myo7a^fl/fl^Myo15-cre^+/−^Tecta^−/−^* mice (*F*) at −84 mV in the presence of 5 mM intracellular BAPTA. (*G* and *H*) Maximal size of the MET current (*G*) and *P_open_* (*H*) recorded from the IHCs of both genotypes. Data are plotted as mean ± SD. (*I* and *J*) SEM images showing the hair bundle structure of IHC from P30 to P37 control (*I*) and *Myo7a^fl/fl^Myo15-cre^+/−^Tecta^−/−^* (*J*). *Bottom* panels: higher magnification images. (*K*) Height of the three rows of stereocilia in the IHCs of P28 to P37 mice from both genotypes.

Considering the changes in the MET current recorded from mature OHCs of *Myo7a^fl/fl^Myo15-cre^+/−^Tecta^−/−^* mice (Fig. 3), we tested whether IHCs were equally affected. MET currents in IHCs were measured using 1 mM intracellular EGTA and at a membrane potential of −84 mV. Since the taller hair bundles of IHCs lacking MYO7A could be more easily damaged by the fluid jet stimulation, their displacement required the use of subsaturating stimuli at first, which were then gradually increased until the maximal current was achieved (*Materials and Methods*). The size of the MET current elicited from P21 to P22 apical coil IHCs of *Myo7a^fl/fl^Myo15-cre^+/−^Tecta^−/−^* mice started to be significantly reduced compared to that of control cells (*P* < 0.0001, *t* test, Fig. 5 *A*–*C*). However, as also shown for the OHCs (Fig. 3), the resting *P_open_* of the IHC MET channels was comparable between the two genotypes (*P* = 0.4846, *t* test, Fig. 5*D*). In 5 mM intracellular BAPTA (Fig. 5 *E*–*H*), the larger resting *P_open_* of the MET channel was again indistinguishable between the two genotypes (*P* = 0.2341, *t* test, Fig. 5*H*), despite the significantly reduced size of the MET current in the IHCs from *Myo7a^fl/fl^Myo15-cre^+/−^Tecta^−/−^* mice (*P* = 0.0068, *t* test, Fig. 5*D*), further supporting the finding that the tip links are tensioning the functional MET channels. Despite the loss of the MET current, the height of the IHC hair bundles at around P30 was still indistinguishable between the two genotypes (*P* = 0.9488, two-way ANOVA, Fig. 5 *I*–*K*). The width of the IHC hair bundles was also not significantly different between the two genotypes (*SI Appendix,* Fig. S6*B*).

### Transcriptional Changes in *Myo7a*-Deficient Mice.

To understand the transcriptional changes potentially induced by the knockout of *Myo7a* in the cochlea, we performed RNA-sequencing at P15 and P30, which is before and after the loss of hair-cell function, respectively. For these experiments we compared the auditory sensory epithelium of the original control (*Myo7a^fl/fl^*) and *Myo7a^fl/fl^Myo15-cre^+/−^* mice to avoid detecting gene changes associated with the loss of *Tecta*.

**Fig. 6.**
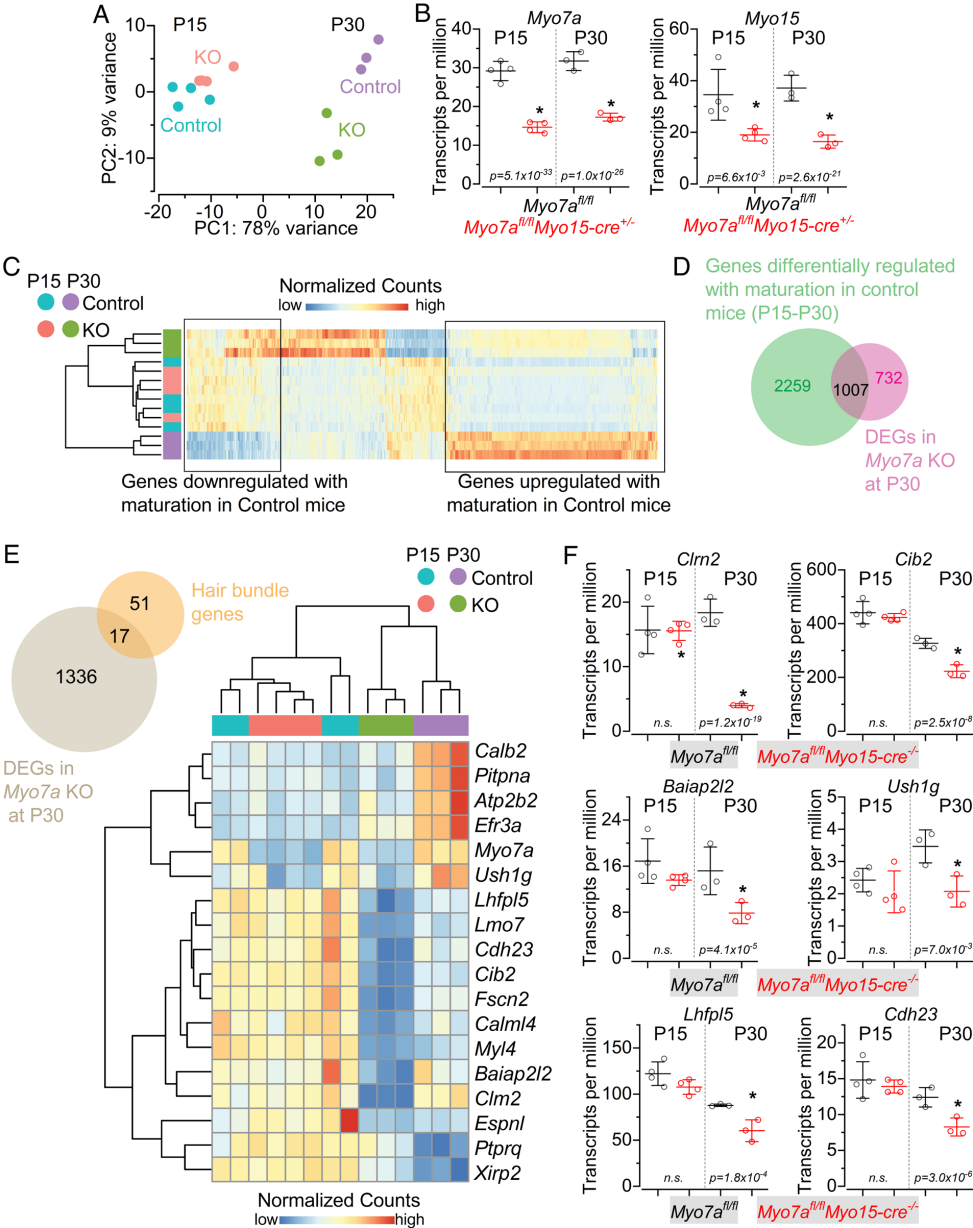
RNA-sequencing analysis in *Myo7a*-deficient mice. (*A*) PCA of the top 2,000 most variable genes in each cDNA library for *Myo7a^fl/fl^* and *Myo7a^fl/fl^Myo15-cre^+/−^* at P15 and P30. (*B*) Transcripts per million from RNA-sequencing libraries for *Myo7a* and *Myo15* at both ages and genotypes. (*C*) Heatmap of differentially expressed genes between control and *Myo7a^fl/fl^Myo15-cre^+/−^* mice at P30. Unsupervised hierarchical clustering on the side shows that P30 *Myo7a^fl/fl^Myo15-cre^+/−^* mice are more similar to P15 mice of both genotypes than to P30 control mice. (*D*) Venn diagram showing overlap between genes whose expression is regulated during cochlear maturation and the genes whose expression changes in *Myo7a^fl/fl^Myo15-cre^+/−^* mice at P30. (*E*) Transcripts per million from RNA-sequencing libraries for six of the identified genes that are downregulated in *Myo7a^fl/fl^Myo15-cre^+/−^* mice at P30. (*F*) Transcripts per million from RNA-sequencing libraries for six of the genes listed in panel *E* at P15 and P30 for both genotypes. Statistical values (*B* and *F*) are from the *P*-value adjusted Deseq2 differential analysis (Log2 fold change > 0.5, lfcse < 0.5, *P*-adjusted value < 0.01). RNA-sequencing data have been deposited in GEO (GSE246143); see *Data, Materials, and Software Availability statement*.

At P15, principal component analysis (PCA) showed similar values between the samples from the two genotypes (Fig. 6*A*). In line with the PCA results, differential expression analysis only revealed 12 differentially expressed genes (DEGs, *SI Appendix,* Table S1), including about 50% decrease in expression of *Myo7a* in *Myo7a^fl/fl^Myo15-cre^+/−^* mice compared to control *Myo7a^fl/fl^* (Fig. 6*B*). A residual expression of MYO7A in *Myo7a*-deficient mice (24% remaining compared to controls) was confirmed at the protein level by performing western blot from the mature cochlea (*SI Appendix,* Fig. S9). Considering that the *Myo15*-cre mouse line is specific for the sensory hair cells (11), as also demonstrated by our immunostaining experiments (Fig. 2), the residual MYO7A in *Myo7a*-deficient mice is most likely originating from other sources. Single-cell RNA sequencing from the mouse cochlea (30, 31) and data from the human protein atlas (https://www.proteinatlas.org/ENSG00000137474-MYO7A) have shown *Myo7a* expression in different nonsensory cell types within the auditory epithelia, as well as cochlear macrophages (32). *Myo15* was also significantly downregulated in *Myo7a^fl/fl^Myo15-cre^+/−^* mice (~1.8 decrease in expression: Fig. 6*B*), which is due to the heterozygosity of the *Myo15*-cre mouse (11). Despite this downregulation due to its heterozygous expression, MYO15 was present in the hair bundles of OHCs even at older ages (P31: *SI Appendix,* Fig. S10) and did not affect either the biophysical properties of adult IHCs and OHCs or hearing function (23). These results indicate that at P15, *Myo7a^fl/fl^Myo15-cre^+/−^* mice did not show a significant change in the overall transcriptional landscape. By contrast, at P30, in addition to *Myo7a* and *Myo15,* we observed significant transcriptional changes with 1,005 genes upregulated and 734 genes downregulated (Fig. 6 *A* and *C* and *SI Appendix,* Table S2).

We next sought to understand how many of these differentially expressed genes were normally up or downregulated during cochlear maturation. To identify the changes in gene expression occurring during posthearing maturation between P15 and P30, we performed differential expression analysis between the cochlea of control mice (*Myo7a^fl/fl^*) from both ages. As expected, 92% of the variance in the data could be explained by the age difference, with 3,266 genes differentially expressed (*SI Appendix*, Fig. S11 and Table S3). Of these, 30% of the genes (1,007/3,266) were affected in *Myo7a^fl/fl^Myo15-cre^+/−^*. Among the DEGs at P30, 58% of them (1,007/1,739) were up- or down-regulated between P15 and P30 (Fig. 6*D*).

### Effect of *Myo7a* Knockout on Hair Bundle Gene Expression.

To investigate whether the reduction in MYO7A (>87%) in the hair cells influenced the expression of other stereociliary bundle genes, we compiled a list of hair bundle/stereocilia-associated genes from the literature (*SI Appendix,* Table S4) and compared them to the DEGs that we identified. At P15, we did not find any transcriptional changes in *Myo7a^fl/fl^Myo15-cre^+/−^* mice, indicating that the strongly reduced or absent MYO7A (Fig. 1) did not directly alter the expression of the hair bundle proteins, which agrees with the normal MET current (Fig. 3) and hearing function (Fig. 1) up to about P20. However, at P30, we found that out of the 64 identified genes from the literature (*Myo7a* was removed from the list), 16 of them (~25%) overlapped with the differentially expressed genes in *Myo7a^fl/fl^Myo15-cre^+/−^* mice (Fig. 6*E*). Of these 16 genes, 14 were downregulated, suggesting a progressive loss of the hair-bundle gene-expression program in *Myo7a^fl/fl^Myo15-cre^+/−^* mice, most likely as a result of an indirect compensatory mechanism caused by the missing MYO7A. Most of these genes have been shown to be essential for mechanoelectrical transduction, including *Clrn2, Cib2*, *Baiap2l2, Ush1g, Lhfpl5,* and *Cdh23* (Fig. 5*F*). Immunostaining experiments showed that the percentage of transducing stereocilia with BAIAP2L2 puncta (*SI Appendix,* Fig. S10) was significantly reduced in the OHCs of *Myo7a^fl/fl^Myo15-cre^+/−^* mice (39 ± 12%) compared to controls (95 ± 5%, *P* < 0.0001, *t* test). Despite the >87% reduction in MYO7A in the stereocilia, the scaffolding protein USH1C (Harmonin), which together with MYO7A and USH1G (Sans) constitutes the major protein complex in the upper tip-link density required for their tensioning (33), was present in the OHC hair bundles of *Myo7a^fl/fl^Myo15-cre^+/−^* mice (*SI Appendix*, Fig. S12). This finding further supports previous observations showing that a reduced level of MYO7A, or the presence of MYO7A without motor activity (*Shaker 1* mouse), does not affect the localization of both USH1C and USH1G at the upper tip-link density (UTLD) (10, 15). Of the 1,007 genes downregulated in P30 *Myo7a^fl/fl^Myo15-cre^+/−^* mice, 71% of them (713) were upregulated between P15 and P30 in control *Myo7a^fl/fl^* mice, suggesting that the reduced *Myo7a* expression affected the general functional maturation and maintenance of the hair cells.

### Noise Exposure Exacerbates the Progression of Hearing Loss and Hair Bundle Disorganization in *Myo7a*-Deficient Mice.

**Fig. 7.**
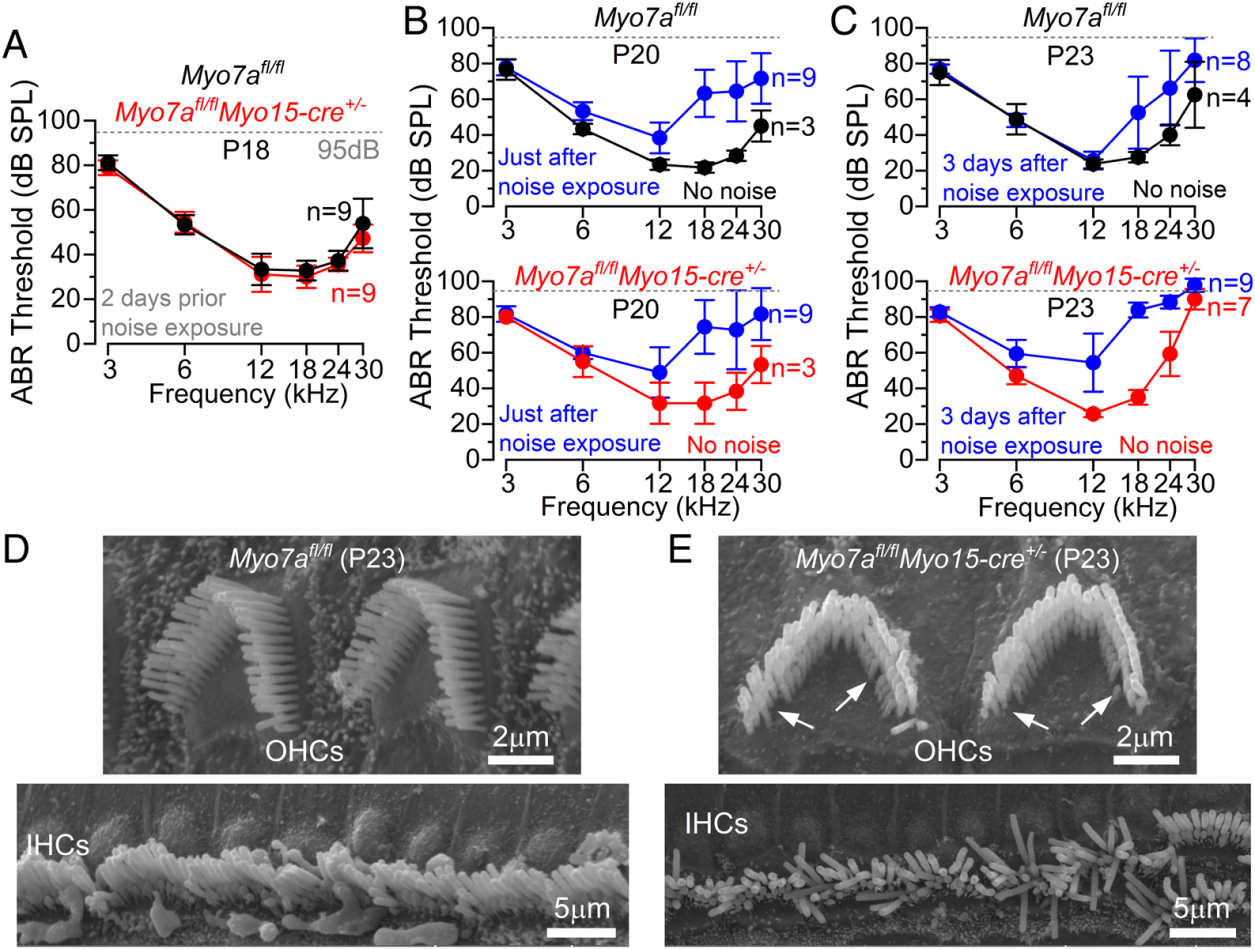
Noise exposure exacerbates hearing loss in *Myo7a*-deficient mice. (*A–C*) Average ABR thresholds recorded from *Myo7a^fl/fl^* (black) and *Myo7a^fl/fl^Myo15-cre^+/−^* mice (red). Recordings were performed at: P18, 2 d prior to noise exposure (*A*); P20, just after noise exposure (*B*); P23, 3 d after noise exposure (*C*). Noise exposed recordings are shown in blue. Number of mice tested is shown next to the data. The dashed line: upper threshold limit of our system, 95 dB. (*D* and *E*) SEM images showing the OHC and IHCs hair bundle structure of P23 *Myo7a^fl/fl^* (*D*) and *Myo7a^fl/fl^Myo15-cre^+/−^* (*E*) mice. Arrows in panel *E* indicate missing stereocilia in *Myo7a^fl/fl^Myo15-cre^+/−^* mice.

To test whether the hair bundles of *Myo7a*-deficient mice are more susceptible to insults due to the dysregulation of many stereociliary proteins, we exposed control *Myo7a^fl/fl^* and *Myo7a^fl/fl^Myo15-cre^+/−^* mice to noise for 2 h (bandwidth of 1 to 16 kHz and delivered at an intensity of 96 to 97 dB SPL), which causes a temporary threshold shift (TTS) of the ABR thresholds. ABR thresholds to pure tones (3 to 30 kHz) were measured 2 d before (P18) and immediately after noise exposure at P20, and then again at P23 to look for recovery from TTS in auditory function (Fig. 7 *A*–*C*). In control mice, the elevated ABR thresholds returned to near-normal levels after just 3 d following the noise insult (Fig. 7 *B* and *C*, *Upper*). By contrast, ABR thresholds in P23 *Myo7a^fl/fl^Myo15-cre^+/–^* mice did not recover (Fig. 7 *B* and *C*, *Lower*), showing that noise exposure exacerbated the progression of hearing loss. Scanning electron microscopy images highlighted that noise exposure also exacerbated the damage to the hair cells (Fig. 7 *D* and *E*), with an average of two OHCs lost within the field of view (~60 µm) and severe damage of the stereocilia, especially those of the IHCs (Fig. 7 *D* and *E*), which was only observed from around 2 mo of age onward (*SI Appendix,* Fig. S1).

**Fig. 8.**
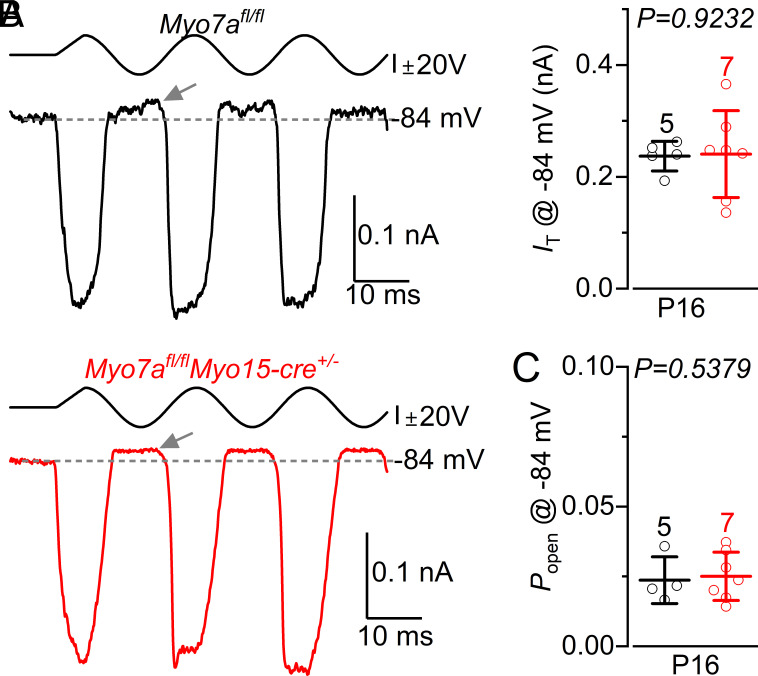
Mechanoelectrical transduction in IHCs from *Myo7a^fl/fl^Myo15-cre^+/−^* mice. (*A*) MET currents recorded from IHCs of P16 control and *Myo7a^fl/fl^Myo15-cre^+/−^* mice at −84 mV (1 mM intracellular EGTA). Arrows indicate the presence of the resting MET current. (*B* and *C*) Average peak- to-peak MET current (*B*) and resting MET current *P_o_* (*C*) from IHCs of control (*n* = 5, two mice) and *Myo7a^fl/fl^Myo15-cre^+/−^* (*n* = 7, four mice) mice (mean ± SD). Statistical values: *t* test.

In a previous study we showed that the size of the MET current as well as the resting *P_open_* in IHCs from *Myo7a^fl/fl^Myo15-cre^+/−^* mice were already reduced by P16 compared to controls (23). Considering that our current data show that ABRs are indistinguishable between *Myo7a^fl/fl^* and *Myo7a^fl/fl^Myo15-cre^+/−^* at least up to P20 (Fig. 1), we wondered whether the early abnormal MET current in IHCs from *Myo7a-*deficient mice (23) was due to their bundle being more prone to damage due to the artificial bundle stimulation with the fluid jet. We tested this hypothesis by performing MET current recordings from P16 *Myo7a^fl/fl^* and *Myo7a^fl/fl^Myo15-cre^+/−^* by using the same more careful approach (*Materials and Methods*) applied to the IHC recordings (Figs. 3 and 4). We found that the size and resting MET current recorded from IHCs of P16 *Myo7a^fl/fl^Myo15-cre^+/−^* mice was not significantly different compared to control cells (Fig. 8), which agrees with the normal ABR thresholds up to at least P20 (Fig. 1).

## Discussion

Here, we show that in mature cochlear hair cells, MYO7A is required for maintaining the morphological and functional integrity of the stereociliary bundles housing the MET complex. The reduction in MYO7A (up to 87 to 98%) in hair cells from posthearing mice led to the loss of their function, which was caused by the progressive reduction of the MET current in both OHCs and IHCs without affecting, at least initially, the morphology of their hair bundles. However, the resting open probability of the MET channel and its dependence on intracellular Ca^2+^ were not affected in hair cells from *Myo7a*-deficient mice. Cochlear RNA-sequencing analysis from *Myo7a-*deficient mice highlighted the downregulation of several genes known to be essential for mechanoelectrical transduction. These compensatory transcriptional changes are likely to underlie the reduced stiffness of the OHC hair bundles and the increased susceptibility of the hair cells to noise damage in *Myo7a*-deficient mice. Although the >87% reduction of MYO7A in the stereocilia did not affect the size of the MET current at P20, it made the hair bundles more susceptible to damage from loud sounds or artificial stimulation. We propose MYO7A plays a role in providing morphological and functional stability to the stereociliary bundles of mature cochlear hair cells, essential for maintaining their integrity over a lifetime of acoustic stimuli.

### Role of MYO7A in Hair-Cell Mechanoelectrical Transduction.

Usher type 1 syndrome proteins MYO7A, USH1C (Harmonin), and USH1G (Sans) are believed to form a tripartite complex at the UTLD, which is localized at the insertion of the tip links to the side of the taller stereocilia (10, 33, 34). The adaptor proteins USH1C and USH1G have been associated with the scaffolding linking the upper end of the tip link, which is formed by CDH23 (6, 7), with the F-actin filament of the stereocilia via the motor protein MYO7A (10, 11). Early studies using constitutive knockout mice have shown that the absence of MYO7A leads to early and severe morphological defects in the stereociliary bundles of the hair cells (21, 22). In addition to the morphological defects, electrophysiological experiments have shown that MYO7A is essential for setting the resting open probability of the MET channels by controlling the tip link tension (21). This resting open probability is crucial for proper sound transduction because it sets the mechanical sensitivity of the MET channel (35). However, these initial physiological studies may have been biased by secondary effects caused by the disorganized hair bundles, and by the presence of an anomalous MET current that can be activated even in the absence of tip-links (36, 37). A recent study from the immature prehearing cochlea has highlighted that hair cells express multiple isoforms of MYO7A, with the canonical isoform MYO7A-C expressed in all IHCs, while in OHCs its expression decreases from the apex to the base of the cochlea (15). Deletion of the canonical MYO7A isoform (*Myo7a-ΔC* mouse) leads to a reduction of MYO7A in the stereocilia of prehearing IHCs, but less in the OHCs located in the cochlear apex (15). Despite the reduction in MYO7A in both cell types, IHCs from *Myo7a-ΔC* mice, but not OHCs, exhibited a strongly reduced resting open probability of the MET channel (15). The authors have proposed that alternative MYO7A isoforms, such as MYO7A-S, could be expressed in OHCs and fulfill a similar role as MYO7A-C in the IHCs. Although this is an attractive hypothesis considering the distinct MET channel properties between prehearing IHCs and OHCs, including calcium permeability (38) and resting open probability (29), there is no experimental evidence supporting it.

In the present study, we found that the reduced expression (>87%) of MYO7A in both IHCs and OHCs at P20 had no immediate effects on the MET current and hearing function in *Myo7a*-deficient mice. However, hearing thresholds of *Myo7a*-deficient mice older than P20 rapidly increased despite no apparent further reduction in MYO7A, which was mirrored by a progressive loss of the MET current in both hair cell types. Despite the reduced MET current, the resting MET channels of both IHCs and OHCs retained a normal resting open probability and calcium sensitivity in *Myo7a*-deficient mice. These findings seem inconsistent with the idea that MYO7A is responsible for controlling the resting MET current. Since the tip links are required to be under tension at rest to maintain the high sensitivity of the MET apparatus, another of the several myosin motors expressed in hair cells or a yet unknown mechanism is likely to be involved. However, we cannot exclude the possibility that residual MYO7A molecule, the detection of which is below the sensitivity of our system, could still be present at the stereocilia 3 to 4 wk following the cre-dependent recombination starting at P3 (11), and be sufficient to maintain the tip-link resting tension of the remaining active MET channels.

### MYO7A Is Required for the Structural and Functional Integrity of the Hair Cell Stereociliary Bundles.

In hair cells, MYO7A appears to have a slow turnover since, despite the Cre-mediated deletion of *Myo7a* by the *Myo15* promoter occurring at about P3 to P4 in apical hair cells (11), it was still detectable in the hair cells a week later. This delay allowed the stereociliary bundle of both OHCs and IHCs to develop normally in *Myo7a*-deficient mice. The staircase structure of the hair bundle remained indistinguishable from that of control cells at least up to P37 (see also 23), by which time mice are already deaf. Despite the normal-looking hair bundles of OHCs from P30 *Myo7a*-deficient mice, their steady-state stiffness was about half that of control cells. Tip link tensioning has been shown to contribute about a third of the hair bundle stiffness in mouse OHCs (39). Considering that at P30 the MET current in *Myo7a*-deficient mice is likely to be very small despite the presence of the tip links in hair cell bundles, it is possible that the links no longer exert tension on the “nonfunctional” MET complex, thus contributing to the reduced hair bundle stiffness. Moreover, by P30 the expression of many genes essential for mechanoelectrical transduction was affected, possible further contributing to the reduced bundle stiffness.

The dysregulation of several key genes in *Myo7a*-deficient mice is likely to affect the structural integrity of the hair bundles that are continuously subjected to wear and tear due to sound stimulation. Indeed, the hair bundles of *Myo7a*-deficient mice progressively deteriorate and are no longer present by about 6 mo of age. This morphological degradation of the stereocilia is consistent with the structural role attributed to MYO7A from previous studies using constitutive knockout mice (21, 22). Indeed, we found that IHCs with a more fragile hair bundle structure when MYO7A is largely reduced (87%) are more likely to lose their resting MET current when strongly stimulated (23), but not when stereocilia are more carefully displaced. Further evidence for this structural role for MYO7A comes from noise exposure experiments, where noise-induced disruption of the stereocilia lacking MYO7A accelerates the progressive loss of hearing function.

MYO7A, like the several other unconventional myosin motors expressed in the mammalian cochlea (e.g., MYO3A, MYO6, and MYO15A), is essential for the development and maintenance of the stereociliary bundles (40, 41). The classical role of these unconventional myosins is to assemble and shape stereocilia architecture by using their motor activity to deliver structural and actin-regulatory cargo to the stereocilia. MYO7A has been shown to localize several key bundle proteins, including the tip-link component PCDH15 (8), the barbed-end capping protein Twinfilin 2 (42, 43), and the ankle-link components ADGRV1 (USH2C) and Usherin (USH2A) (44, 45). The disparity of activities suggested for MYO7A, including its molecular interaction with many other bundle proteins (41), highlight the complex role fulfilled by this unconventional myosin not only in establishing the hair bundles (22), but also in maintaining their structural and functional integrity in the adult cochlea that is continuously subjected to sound stimulation. Over time, the reduced level of MYO7A leads to hair cell degeneration, as also demonstrated for other key molecules involved in mechanoelectrical transduction such as TMC1 (46) and MYO6 (47).

## Materials and Methods

### Ethics Statement and Animal Strains.

All experiments involving mice were licensed by the UK Home Office. See *SI Appendix*, *Methods*.

### In Vivo Auditory Function and Noise Exposure.

ABRs and DPOAEs were performed as previously described (28). Some mice were noise-exposed to 1 to 16 kHz at 96 to 97 dB SPL for 2 h, which causes temporary ABR threshold shift. See *SI Appendix*, *Methods*.

#### Electrophysiology and Hair Bundle Stimulation.

Electrophysiological recordings were performed using an Optopatch amplifier. The hair bundles of hair cells were displaced using a fluid jet from a pipette (28, 29). See *SI Appendix*, *Methods*.

#### Immunofluorescence Microscopy and Scanning Electron Microscopy (SEM).

Cochleae were fixed with 4% PFA (immuno) or 2.5% glutaraldehyde (SEM). See *SI Appendix*, *Methods*.

### RNA Isolation, Library Preparation for RNA-Sequencing, and Analysis.

Tissue was frozen in liquid nitrogen after dissection and then thawed on ice before RNA extraction. For additional information about RNA isolation, library preparation, and analysis (**SI Appendix*, Methods*).

### Statistical Analysis.

Statistical analysis is indicated throughout the text. *P* < 0.05 was selected as the criterion for statistical significance. Data are shown as means ± SD. Animals of either sex were randomly assigned to the different experimental groups.

## Supplementary Material

Appendix 01 (PDF)

## Data Availability

RNA-sequencing data have been deposited in GEO (GSE246143) (48).
