# Supplementary material for: MYO7A is required for the functional integrity of the mechanoelectrical transduction complex in hair cells of the adult cochlea
Source: Proc Natl Acad Sci U S A. 2025 Jan 2;122(1):e2414707122. doi: 10.1073/pnas.2414707122 (PMC11725811; doi:10.1073/pnas.2414707122)
Supplement: Supplementary file 1 — Appendix 01 (PDF) [file pnas.2414707122.sapp.pdf]

PNAS

Supporting Information for

MYO7A is required for the functional integrity of the mechanoelectrical transduction complex in hair cells of the adult cochlea.

Anna Underhill, Samuel Webb, Fiorella C. Grandi, Jing-Yi Jeng, Jacques B. de Monvel, Baptiste Plion, Adam J. Carlton, Ana E. Amariutei, Niovi Voulgari, Francesca De Faveri, Federico Ceriani, Mirna Mustapha, Stuart L. Johnson, Saaid Safieddine, Corné J. Kros, Walter Marcotti

#Corresponding author:
Walter Marcotti (w.marcotti@sheffield.ac.uk).

This pdf file includes:

Supporting Methods – Pgs. 2-9
Figures S1-S12 – Pgs. 10-21
Supporting References – Pg. 22
Supporting Tables S1 – Pg. 23
Supporting Tables S2 – Pgs. 24-43
Supporting Tables S3 – Pgs. 44-76
Supporting Tables S4 – Pg. 77
Supporting Tables S5 – Pgs. 78-79

Supporting Information Text

Materials and Methods

Ethics statement

All experiments involving mice were licensed by the UK Home Office under the Animals (Scientific Procedures) Act 1986 (PPL_PCC8E5E93) and were approved by the University of Sheffield Ethical Review Committee (180626_Mar). For *in vivo* auditory brainstem responses (ABRs) and distortion product otoacoustic emissions (DPOAEs) mice were anesthetized using intraperitoneal injection of ketamine (100 mg/kg body weight, Fort Dodge Animal Health, Fort Dodge, USA) and xylazine (10 mg/kg, Rompun 2%, Bayer HealthCare LLC, NY, USA). Following the onset of anaesthesia and the loss of the retraction reflex to a toe pinch, mice were placed on a thermal mat in a soundproof chamber for ABR or DPOAE experiments. At the end of the ABR or DPOAE recordings, mice were either culled by cervical dislocation or recovered from anesthesia with an intraperitoneal injection of atipamezole (1 mg/kg) and returned to their cage, placed on a thermal mat and monitored over the following 2-5 h. For *ex vivo* experiments mice were killed by cervical dislocation followed by decapitation.

Animal strains

For the conditional knockout mice (*Myo7a*$^{fl/fl}$*Myo15-cre*$^{+/-}$), the targeted *tm1a* allele for *Myo7a* (*Myo7a*$^{tm1a(EUCOMM)Wtsi}$ allele ID: 4431921) was generated by the Mouse Genetics Programme at the Wellcome Trust Sanger Institute (Cambridge, UK). For *Myo7a* the critical exons 10 and 11 were floxed and the *tm1c* allele was obtained by crossing the *tm1a* mouse to a FLPeR carrying mouse (Rosa26^{Fki}). The *tm1d* alleles, which were used for the experiments, were obtained by crossing the *tm1c* mouse (*Myo7a*$^{fl/fl}$) with the *Myo15*-cre mice. For experiments, cre-negative littermate control (referred to the manuscript as *Myo7a*$^{fl/fl}$) and knockout mice (*Myo7a*$^{fl/fl}$*Myo15-cre*$^{+/-}$) were used. *Myo7a*$^{fl/fl}$*Myo15-cre*$^{+/-}$ mice were crossed with *Tecta* knockout mice (1) to generate a double knockout mouse for both *Myo7a* and *Tecta*, and referred to the manuscript as: *Myo7a*$^{fl/fl}$*Tecta*$^{-/-}$ (control) and *Myo7a*$^{fl/fl}$*Myo15-cre*$^{+/-}$*Tecta*$^{-/-}$ (knockouts).

Distortion product otoacoustic emissions

Distortion product otoacoustic emissions (DPOAEs) experiments, which are a product of cochlear amplification caused by OHC electromotility during acoustic stimulation of their hair bundles, were performed in a soundproof chamber (MAC-3 Acoustic Chamber, IAC Acoustic, UK) and were recorded at 2f1-f2 in response to primary tones f1 and f2, where f2/f1 = 1.2. The f2 level (L2) was set from 20 to 80 dB (the maximum level set for our system) in 10 dB increments, and the f1 level (L1) was set equal to L2. Frequency pairs of tones between f2 = 6.5 kHz and f2 = 26.3 kHz were

delivered into the left ear canal of mice by using a coupler connected to two calibrated loudspeakers (MF1-S, Multi Field Speaker, Tucker-Davis Technologies, USA). The response signal was averaged over 500 repetitions. The emission signals were recorded by a low-noise microphone (ER10B+: Etymotic Research Inc, USA) connected to the same ear coupler mentioned above. Experiments were performed using BioSigRZ software driving an RZ6 auditory processor (Tucker-Davis Technologies). DPOAE thresholds were defined by the minimal sound level where the DPOAEs were above the standard deviation of the noise using a custom-made software.

Auditory brainstem responses

Male and female mice were placed on a heated mat (37ºC) inside the same soundproof chamber (MAC-3) with the animal's pinna positioned 10 cm from the loudspeaker (MF1-S, Multi Field Speaker, Tucker-Davis Technologies, USA), which was calibrated with a low-noise microphone probe system (ER10B+, Etymotic, USA). Two subdermal electrodes were placed under the skin behind the pinna of each ear (reference and ground electrode), and one electrode half-way between the two pinna on the vertex of the cranium (active electrode) as previously described (2). Experiments were performed using a customized software (3) driving an RZ6 auditory processor (Tucker-Davis Technologies). ABR responses were measured for white noise clicks and pure tone stimuli at frequencies of 6, 12, 18 and 24 kHz. ABR thresholds were defined as the lowest sound level where any recognisable feature of the waveform was visible. Stimulus sound pressure levels were up to 95 dB SPL, presented in steps of 5 dB SPL (average of 256 repetitions). Tone bursts were 5 ms in duration with a 1 ms on/off ramp time presented at a rate of 42.6/sec.

Noise exposure

For noise exposure, each awake and unrestrained mouse was housed within a wire cage suspended at the centre of a custom-made wooden box (4), with the following dimensions: base 58.4 cm^2; top: 45.7 cm^2; height front: 59.5 cm; height back: 65 cm. These dimensions ensure that no two sides of the wooden box are parallel. To deliver the stimuli, 1-16KHz broadband noise was generated using Audacity software (Audacity 3.2.1). The stimuli were amplified to 97dB SPL using a W audio TPX 650 amplifier connected to a U-Phoria UMC22 audio interface played through a speaker (Beyma TPL200/S) positioned at the top of the soundproof box. Noise calibration to the target SPL and frequency was performed immediately before each noise exposure session using a low-noise microphone probe system (ER10B+, Etymotic, USA) connected to an oscilloscope (Picoscope 2000 series, Pico Technology). Measuring across the wire cage where the mice were kept, the sound pressure varied by less than 2 dB SPL. After calibration, the mice were exposed to the 1–16 kHz at

96-97 dB SPL for 2 h, which causes temporary ABR threshold shift. Throughout the experiments, a camera was used to monitor the mice for any adverse reactions in response to the noise exposure.

Tissue preparation

The cochlear sensory epithelium (organ of Corti) from both male and female mice was dissected out in an extracellular solution composed of (in mM): 135 NaCl, 5.8 KCl, 1.3 $CaCl_2$, 0.9 $MgCl_2$, 0.7 NaH_2PO_4, 5.6 D-glucose, 10 HEPES-NaOH. Amino acids, vitamins and sodium pyruvate (2 mM) were added from concentrates (Thermo Fisher Scientific, UK). The pH was adjusted to 7.48 with 1M NaOH (osmolality ~308 mOsm kg^{-1}). The isolated sensory epithelium was transferred to a microscope chamber, immobilised via a nylon mesh attached to a stainless-steel ring and continuously perfused with the above extracellular solution using a peristaltic pump (Cole-Palmer, UK). The chamber was then mounted on a rotating stage of upright microscopes (Leica DMLFS, Germany; Olympus BX51, Japan) with Nomarski Differential Interference Contrast (DIC) optics (63x or 60x water immersion objective) and 15x eyepieces.

Single-cell electrophysiology

Outer hair cells (OHCs) were studied in acutely dissected organs of Corti with single-cell electrophysiological recordings performed at room temperature (20-24°C) using an Optopatch amplifier (Cairn Research Ltd, UK) as previously described (5,6). All recordings were performed using the above extracellular solution. Recordings were performed using patch pipettes pulled from soda glass capillaries with a typical resistance in extracellular solution of 2-3 MΩ. Patch electrodes were coated with surf wax (Mr Zoggs SexWax, USA) in order to reduce the electrode capacitance. For basolateral membrane recordings, the intracellular solution used in the patch pipette contained (in mM): 131 KCl, 3 $MgCl_2$, 1 EGTA-KOH, 5 Na_2ATP, 5 HEPES-KOH, 10 Na-phosphocreatine (pH was adjusted with 1M KOH to 7.28; 294 mOsm kg^{-1}). The patch pipettes used for recording the MET current contained (in mM): 135 CsCl, 2.5 MgCl2, 1 EGTA-CsOH, 2.5 Na2ATP, 10 sodium phosphocreatine, 5 Hepes-CsOH (pH 7.3). In some experiments, the fast Ca^{2+} buffer 1,2-Bis(2-aminophenoxy)ethane-N,N,N′,N′-tetraacetic acid (BAPTA, Molecular Probes) was used instead of 1 mM EGTA in the Cs-based intracellular solution. When BAPTA was used, the osmolality of the intracellular solution was kept constant by adjusting the concentration of CsCl.

Data acquisition was controlled by pClamp software using a Digidata 1440A (Molecular Devices, USA). Recordings were low-pass filtered at 2.5 kHz (8-pole Bessel), sampled at 5 kHz and stored on a computer for off-line analysis (Clampfit, Molecular Devices; Origin 2021: OriginLab, USA). Membrane potentials under voltage-clamp conditions were corrected off-line for the residual series resistance R_s, which was normally compensated by 80%, and the liquid junction potential (LJP) of –4

mV, which was measured between electrode and bath solutions. For basolateral membrane currents, the holding currents were plotted as zero current to allow a better comparison between recordings. Voltage clamp protocols were performed from a holding potential of −84 mV.

MET current recordings

For mechanoelectrical transducer (MET) current recordings, the hair bundles of the OHCs and IHCs were displaced using a fluid jet from a pipette driven by a 25 mm diameter piezoelectric disc (2,5,7). The pipette was pulled from borosilicate glass to a final overall length of 5.5 cm and contained a normal extracellular solution (see "Tissue preparation"). The fluid jet pipette tip had a diameter of 8-10 μm and was positioned at about 7-8 μm from the hair bundles to elicit a maximal MET current. Mechanical stimuli were applied as 50 Hz sinusoids (filtered at 1 kHz, 8-pole Bessel) with varying saturating driving voltages. For IHCs, the displacement of their taller and less stiff hair bundles required a more careful approach because they were easily damaged, which prevented the measurements of reliable MET currents. This more careful approach required the use of sub-saturating stimuli at first, which were then gradually increased until the maximal current was achieved. Prior to the positioning of the fluid jet by the hair bundles, any steady-state pressure was removed by monitoring the movement of debris in front of the pipette. The use of the fluid jet allows for the efficient displacement of the hair bundles in both the excitatory and inhibitory directions, which is essential to perform reliable measurements of the resting open probability of the MET channels (7-9).

Hair bundle stiffness

For hair bundle displacement imaging, a symmetrical step protocol alternating from positive to negative voltages, with increasing driver voltages (DV) up to ± 1 V in ± 0.1 V increments, was applied to the fluid jet, which was positioned about 7.5 μm from the hair bundles. Hair bundle displacement was imaged by a fast camera (VEO 610L, Phantom) with the Phantom Camera Control software (Phantom). Hair bundle displacement was imaged with a colour depth of 12-bit, using a 480 x 240 (73.28 x 36.64 μm) pixel region of interest at 20000 frames per second (50 μs per frame). Individual frames were converted to a stack, saved as TIFFs and processed using ImageJ. In ImageJ, the total pixels for each frame in the stack were increased in size by a factor of 10 using interpolation. Stacks were resliced along a 1-pixel vertical line drawn across the midpoint of the hair bundle following the fluid jet force displacement. The interpolated reslices produced 2D images where the horizontal direction represents time (x-plane, each pixel/frame = 50 μs) and the vertical direction represents space (y-plane, each pixel = 15.27 nm) along the line drawn (kymograph). In the kymograph, the stereocilia can be visualised as a bright "band" of pixels separate from the background, which when

measuring the grey values for a single frame across the y plane, follow a gaussian distribution, where the brightest pixels represent the central point of the stereocilia. Using this observation, we estimated the position of the stereocilia in the time-series by fitting the grey value bit profile distribution across the y-plane for each frame (63470 per recording) of the kymograph with the following function: $F=I_0+Ae\text{-}((y\text{-}yc)^2)/2w^2$, where I_0 is the offset, A is the amplitude, y is the pixel position in space, yc is the central pixel position and w is half the width of the distribution of grey values. We used the value yc from the fit of every frame as the position of the hair bundle throughout the displacement protocol.

Steady-state hair bundle stiffness was calculated from the linear fluid velocity of the fluid jet, which was calibrated against a carbon fibre of 8 µm in diameter, and by modelling the hair bundles dimension as previously described (7,10). Hair bundle dimensions were estimated with the same upright microscope used to quantify their displacement (Leica DMLFS, Germany) with a 63x water immersion objective, a 15x eyepieces and a calibrated the z-axis (focus). Bundle dimensions were: P20, *Myo7a*$^{fl/fl}$*Tecta*$^{-/-}$ (width 5.6 ± 0.5 µm, height 3.85 ± 0.27 µm, n = 27); P20, *Myo7a*$^{fl/fl}$*Myo15-cre*$^{+/-}$*Tecta*$^{-/-}$ (width 5.5 ± 0.5 µm, height 3.81 ± 0.27 µm, n = 29); P30, *Myo7a*$^{fl/fl}$*Tecta*$^{-/-}$ (width 5.7 ± 0.5 µm, height 3.79 ± 0.28 µm, n = 34); P30, *Myo7a*$^{fl/fl}$*Myo15-cre*$^{+/-}$*Tecta*$^{-/-}$ (width 5.5 ± 0.4 µm, height 3.92 ± 0.28 µm, n = 45.The carbon fibre was placed at the bottom of the recording chamber and at a similar distance (~7.5 µm) from the pipette tip of the fluid jet as for the hair bundle.

Scanning electron microscopy (SEM)

Following the decapitation of the mouse, the inner ear was dissected out and the cochlea was gently perfused with fixative through the round window for about 1 min. Prior to the delivery of the fixative, a small hole was made in the apical portion of the cochlear bone to allow the fixative to flow out from the cochlea. The fixative contained: 2.5% vol/vol glutaraldehyde in 0.1 M Na cacodylate and 2 mM $CaCl_2$ (pH 7.4). Following this initial fixation, the inner ear was transferred in a 1.5 ml conical microtube containing the above fixative and placed on a rotating shaker for 2 h at room temperature. After the 2 h and three washes with 0.1M Na Cacodylate and 2mM $CaCl_2$ buffer, the sensory epithelium of the apical coil was exposed by removing the bone and then immersed in 1% osmium tetroxide in 0.1 M sodium cacodylate buffer for 1 hr. For osmium impregnation, which avoids gold coating, cochleae were incubated for 2 hours in 1% osmium tetroxide in buffer, which was alternated twice with solutions of saturated aqueous thiocarbohydrazide for 20 minutes (the OTOTO technique: 11). The cochleae were then dehydrated through an ethanol series and critical point dried using CO_2 as the transitional fluid (Leica EM CPD300) and mounted on specimen stubs using conductive silver paint (Agar Scientific, Stansted, UK). The apical coil of the sensory epithelium was examined at 10 kV using a Tescan Vega3 LMU scanning electron microscope (Cryo-Electron Microscopy facility,

University of Sheffield) or a FEI Inspect F scanning electron microscope (Sorby Centre for Electron Microscopy, University of Sheffield) in the electron microscopy unit at the University of Sheffield. Three mice were processed for each genotype/experiment. Images were taken from the same region (around 9-12 kHz) used for the electrophysiological recordings. The quantification of the height of the hair bundles in both IHCs and OHCs was performed as previously described (**12**). In brief, each hair bundle was imaged three times with a ±5° tilt between them (-5°, 0 and +5°). Images are taken at a constant magnification (60k for OHCs and 30k for IHCs) and constant working distance (10 mm) across the whole series of data. A single measure (e.g., length from one stereocilium of the tallest row) is taken on a first micrograph and measured again on the corresponding ±5°-tilted repeat micrograph. Tip link quantification was primarily performed between the transducing stereocilia (2nd and 3rd rows), which are better preserved during the processing of the cochleae for SEM. Although less well preserved, we also estimated the presence of tip links between the 1st and 2nd rows of stereocilia.

Immunofluorescence microscopy

Similar to the fixation procedure for SEM, the inner ear was initially perfused through the round and oval windows with a fixative solution (4% paraformaldehyde in phosphate-buffered saline, PBS, pH 7.4) for about 1 min. Following this initial brief fixation, the inner ear was transferred in a 1.5 ml conical microtube and fixed for another 20 min at room temperature with the same fixative solution. Fixed inner ears were then washed three times in PBS for 10 minutes and the cochleae were dissected out using fine forceps and incubated for 1 hr in PBS supplemented with 5% normal goat serum and 0.5% Triton X-100 at room temperature. The samples were then immunolabelled with primary antibodies overnight at 37˚C, washed three times with PBS and incubated with the secondary antibodies for 1 hr at 37˚C. Antibodies were prepared in 1% serum and 0.5% Triton X-100 in PBS. Primary antibodies were: mouse-IgG2a anti-PMCA (1:400, MA3-914, ThermoFisher), rabbit-IgG anti-PMCA2 (1:400, ab3529, Abcam), rabbit-IgG anti-MYO7A (1:500, Proteus Biosciences, 25-6790), rabbit-IgG anti-MYO7A (1:500, DHSB, 138-1-s), rabbit-IgG anti-BAIAP2L2 (1:100, Atlas Antibodies, HPA003043), rabbit-IgG anti-Harmonin (1:200, gift from Saaid Safieddine) and anti-MYO15 (1:400, gift from Dr. Thomas Friedman, NIH, USA). F-actin was stained with Texas Red-X phalloidin (1:400, ThermoFisher, T7471) within the secondary antibody solution. Secondary antibodies were species appropriate Alexa Fluor. Samples were mounted in VECTASHIELD (H-1000). The images from the apical cochlear region (around 9-12 kHz) were obtained with a Zeiss LSM 880 AiryScan equipped with Plan-Apochromat 63x Oil DIC M27 objective (Wolfson Light Microscope Facility at the University of Sheffield). Image stacks were processed with Fiji ImageJ software. At least 3 mice for each genotype were used for each experiment.

For the quantification of myosin VIIA fluorescence intensity, cochlear explants were labelled with both rabbit-IgG anti-MYO7A (1:500, Proteus Biosciences, 25-6790) and rabbit-IgG anti-MYO7A (1:500, DHSB, 138-1-s) to maximise the staining. Images were taken using LSM900 Airyscan (Zeiss, Germany) with a 63x objective or a A1HD25 confocal (Nikon, Japan) with a 60x objective. The selection of points in confocal z-stacks, the computation of normalized local gaussian averages of intensity around these points, and the statistical analysis of the results was performed in MATLAB (The Mathworks). A custom MATLAB interface was used for the display of 3D stacks and manual selection of IHC and OHC points within these stacks. One point was selected at the centre of the cuticular plate, one point at the apical cytoplasmic region, and one point at the basal cytoplasmic region. Three points were selected at each of the two stereocilia levels (stereocilia base or upper tip-link density level): one point at the wedge and two points at the left and right arms of the hair bundle 'W/U'-shape. Two additional points were selected for background estimation, one at a dark position above the cuticular plate, and another one at the centre of the nucleus.

RNA isolation

Tissue was snap frozen in liquid nitrogen after dissection and then thawed on ice in preparation for RNA extraction, with several cochlear tissues combined in one tube. Tissues were homogenized in 350 µl of RLT buffer + DTT using a pestle until the tissue was no longer visible by eye. The homogenized lysate was mixed with 350 µl of 70% ethanol and then applied directly to the Qiagen RNeasy Micro Kit, according to the manufacturer's instructions and RNA was eluted into 15 µl of dH20.

Library preparation and RNA-sequencing

RNA quantity was established using a Nanodrop spectrophotometer and RNA integrity number (RIN) was calculated using a BioAnalyzer. All samples had a RIN score greater than 9.1. Preparation of the mRNA library was performed using poly A enrichment and sequenced on the Illumina NovaSeq sequencer using paired-end 150 bp reads.

RNA-sequencing analysis and differential gene expression calling

The sequencing libraries were processed using the nf-core RNA pipeline (13) (https://nf-co.re/rnaseq/usage) using the standard parameters. Reads were mapped to the mouse genome (mm10). The resulting gene counts were determined using Salmon (14) and used for downstream analysis with DESeq2 (15). Metascape (16), Reactome (17) and StringDB (https://string-db.org/) were used to query for enriched GO terms and pathways in the list of differentially expressed genes.

Western blot

Both cochleae from two mice were dissected in sterile PBS and snap frozen in liquid nitrogen as done for RNA sequencing (see above). Cochleae were then crushed with a sterilised plastic pestle in 100 µl of RIPA buffer (PierceTM, Thermo Fisher #89901) with 1x protease inhibitor cocktail (Roche #11836170001) for 60 seconds. Samples were incubated on ice for a total of 30 minutes followed by centrifugation at 12000 x *g* for 30 minutes at 4ºC. Supernatants were then collected and stored at -20ºC until being run on a 4-15% SDS-PAGE gel (Bio-Rad #4561083). Following 100V transfer onto a PVDF membrane for 1 hour on ice, blots were blocked with 5% low fat skimmed milk powder in TBST (20 mM Tris, 150 mM NaCl, 0.1% Tween 20, pH 7.4) for 1 hour at room temperature. To detect MYO7A, the blots were then incubated with primary antibodies (rabbit-IgG anti-MYO7A 1:1000, Proteus Biosciences, 25-6790) diluted in blocking buffer overnight at 4ºC, rinsed three times and washed three times with TBST for 10 minutes, and then incubated with secondary antibodies (goat anti-rabbit IgG 1:5000, Invitrogen, #31460) for 2 hours at room temperature. Conversely, for the detection of GAPDH, the blots were incubated with HRP-conjugated primary antibodies (HRP-conjugated GAPDH monoclonal antibody 1:10000, Proteintech,#HRP-60004) for 2 hours at room temperature. Following three rinses and three 10-minute washes with TBST, blots were developed with ECL prime western blotting reagent (Cytiva #RPN2232) and imaged on a Gel-Doc XR+ system. Images were captured and analysed using ImageLab software.

Statistical analysis

Statistical comparisons of means were made by Student's two-tailed *t*-test or, for multiple comparisons, analysis of variance (one- or two-way ANOVA followed by a suitable post-test). $P<0.05$ was selected as the criterion for statistical significance. Average values are quoted in text and figures as means ± SD. Animals of either sex were randomly assigned to the different experimental groups. No statistical methods were used to define sample size, which was defined based on our previous work.

***SI Appendix* Fig. S1**

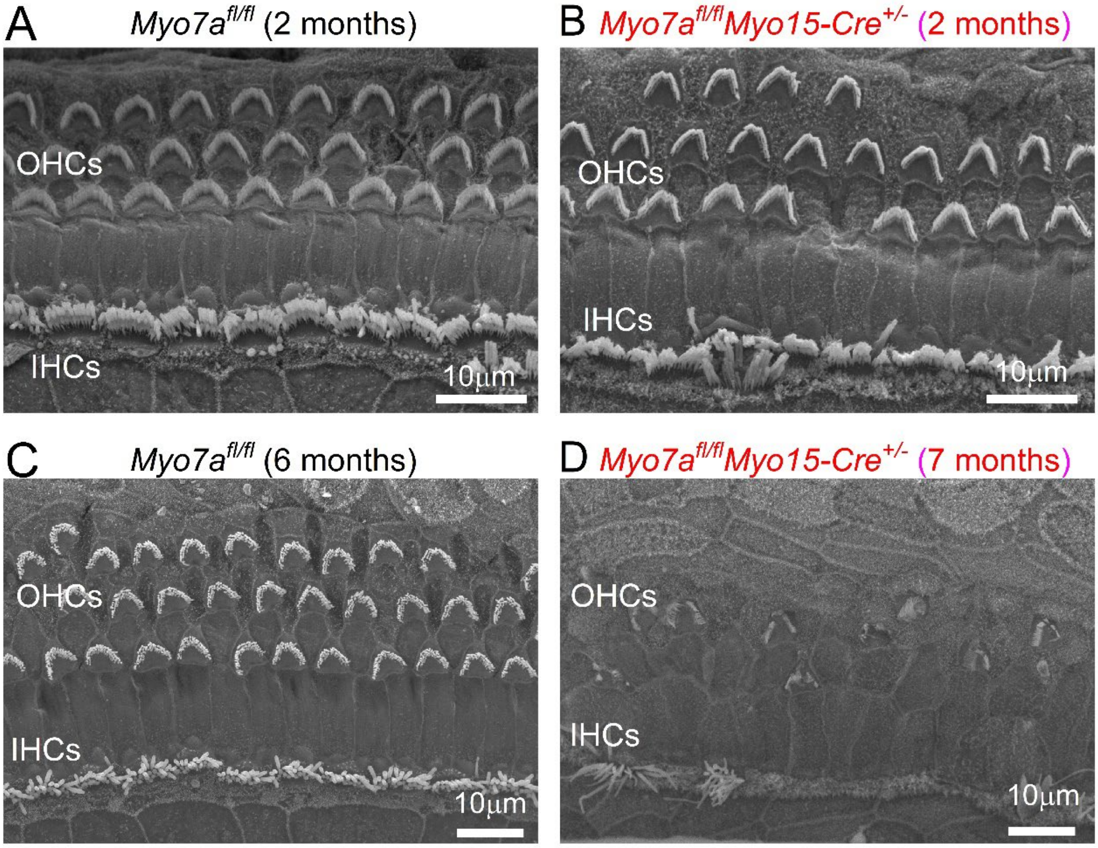

Fig. S1: Hair bundle morphology in ageing *Myo7a$^{fl/fl}$Myo15-cre$^{+/-}$* mice

(***A-D***) Scanning electron microscope (SEM) images showing OHC and IHC hair bundle structure in the apical coil of the cochlea of 2-month and 6-7-month old *Myo7a$^{fl/fl}$* control (***A***: 3 mice; ***C****:* 2 mice, respectively) and *Myo7a$^{fl/fl}$Myo15-cre$^{+/-}$* (***B***: 4 mice; ***D****:* 2 mice, respectively) mice. Note a few missing stereocilia in the 1st and 3rd row of the OHCs from *Myo7a$^{fl/fl}$Myo15-cre$^{+/-}$*. By about 6-7 months of age, the apical coil of the cochlea is almost completely devoid of hair bundles.

SI Appendix Fig. S2

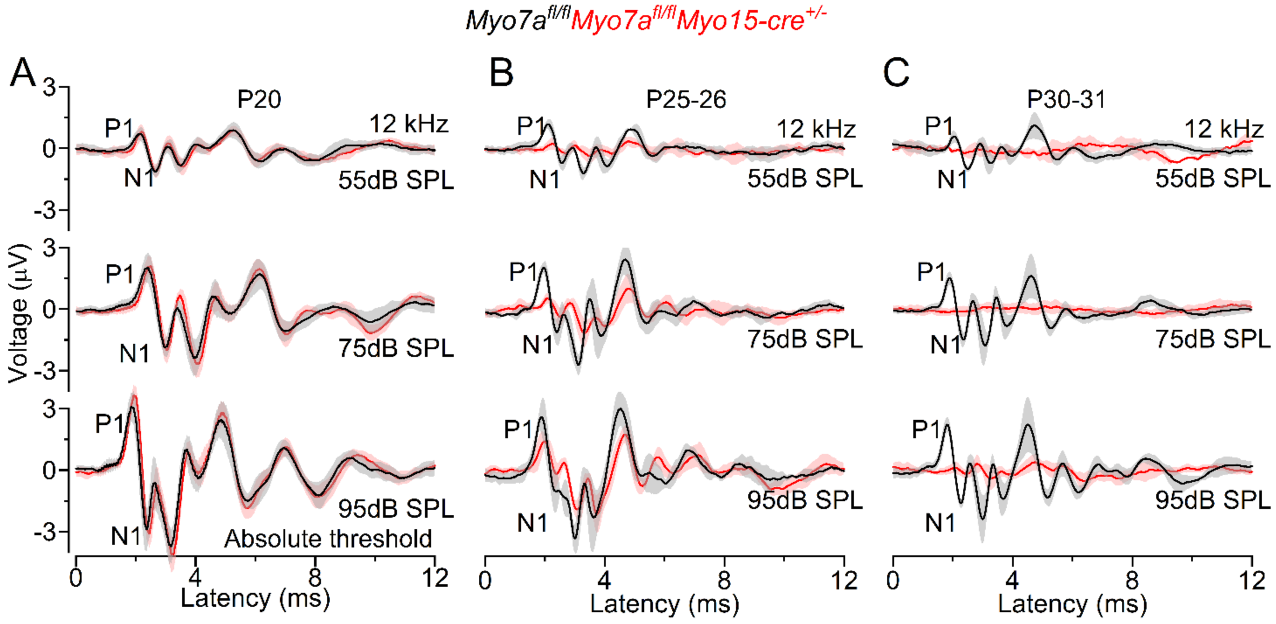

Fig. S2: ABR waveforms evoked in *Myo7a*$^{fl/fl}$ and *Myo7a*$^{fl/fl}$*Myo15-Cre*$^{+/-}$ mice
(***A-C***), Average ABR waveform responses at 12 kHz and 55 dB, 75 dB and 95 dB SPL from control (*Myo7a*$^{fl/fl}$) and littermate *Myo7a*$^{fl/fl}$*Myo15-cre*$^{+/-}$ mice at P20 (***A***), P25-P26 (***B***) and P30-P31 (***C***). Continuous lines: average values (shaded areas represent S.D.). P1 and N1 indicate the positive and negative peaks of wave I. These recordings were included in the average data sets shown in **Fig. 1**.

SI Appendix Fig. S3

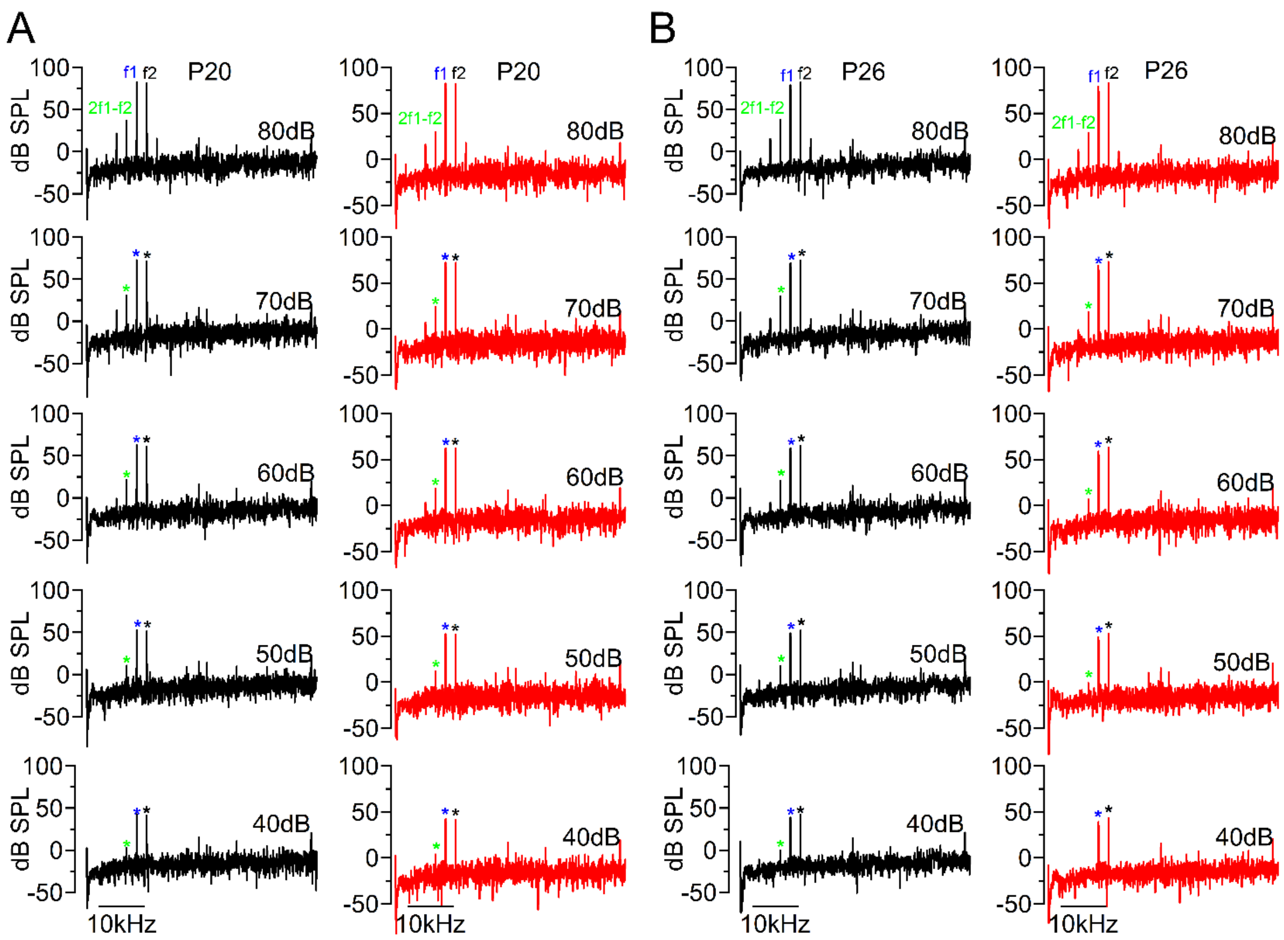

Fig. S3: DPOAE threshold becomes progressively elevated in the absence of *Myo7a*
(A,B) Examples of distortion product otoacoustic emission (DPOAE) waveform responses at around 12 kHz and increasing stimulus intensity (dB SPL) measured from P20 (***A***), P26 (***B***) *Myo7a*$^{fl/fl}$ controls (black) and *Myo7a*$^{fl/fl}$*Myo15-cre*$^{+/-}$ littermate mice (red). These recordings were included in the average data sets shown in **Fig. 1**. The y-axis represents the sound intensity (dB SPL) captured by the microphone inserted into the ear canal. The dB value next below the traces is the sound intensity of f1 and f2. 2f1-f2 represents the distortion product that was measured.

SI Appendix Figure S4

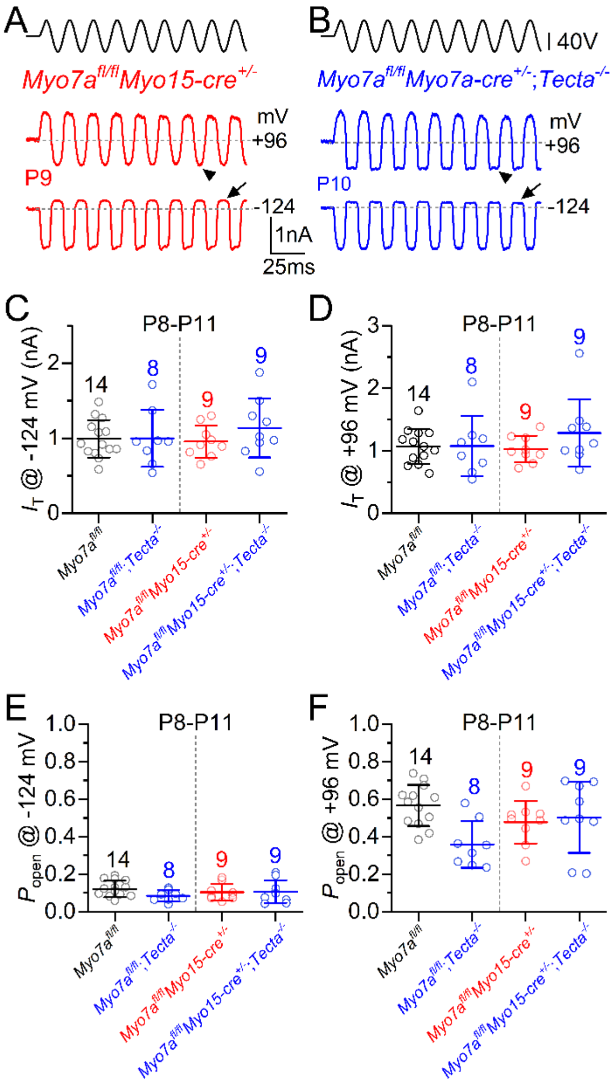

Fig. S4: Mechanoelectrical transducer current properties of OHCs are not affected by the detached tectorial membrane

(***A,B***) Saturating MET currents recorded from apical-coil OHCs of *Myo7a* knockout mice (*Myo7a*$^{fl/fl}$*Myo15-cre*$^{+/-}$: ***A***, P9) and littermates without TECTA (*Myo7a*$^{fl/fl}$*Myo15-cre*$^{+/-}$;*Tecta*$^{-/-}$: ***B***, P10). MET currents were elicited using 50 Hz sinusoidal force stimuli to the hair bundles at membrane potentials of –124 mV and +96 mV. Driver voltage (DV) stimuli to the fluid jet are shown above the traces, with positive deflections of the DV being excitatory. The arrows and arrowheads indicate the closure of the transducer channel in response to inhibitory bundle stimuli at –124 mV and +96 mV, respectively. (***C,D***) Maximal size of the MET current in all strains tested at –124 mV (***C***, $P = 0.4814$, one-way ANOVA) and +96 mV (***D***, $P = 0.4724$, one-way ANOVA). (***E,F***) Resting open probability (P_o) of the MET current in OHCs from the different strains tested at the holding potential of –124 mV (***E***, $P = 0.2532$, one-way ANOVA) and +96 mV (***F***, $P = 0.0858$, one-way ANOVA). The resting current is given by the holding current minus the current present during inhibitory bundle deflection. Overall, the properties of the MET current are not influenced by the presence or absence of either MYO7A or TECTA during pre-hearing ages.

***SI Appendix* Figure S5**

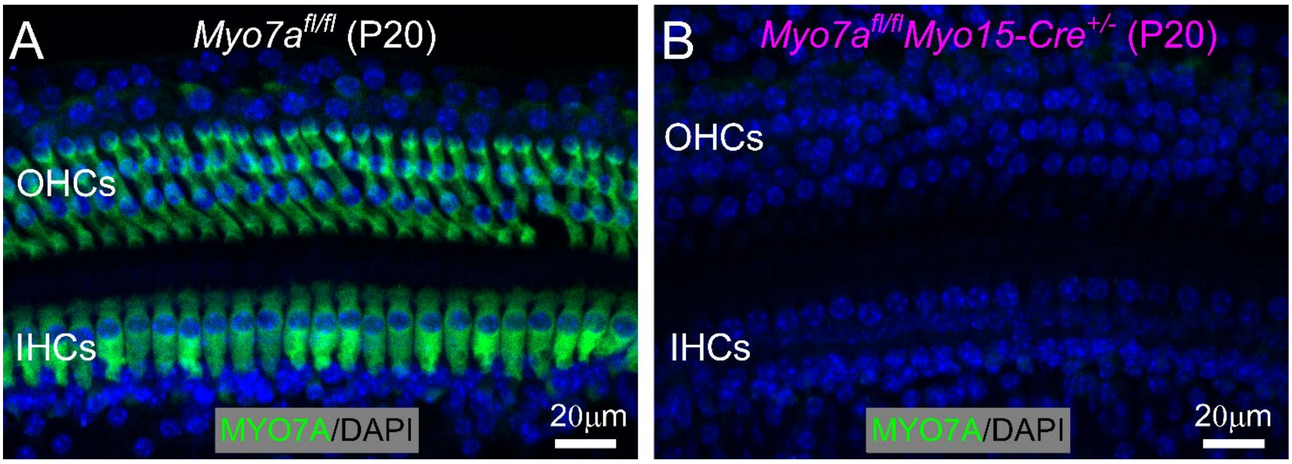

Fig. S5: Expression of MYO7A in the hair cells in *Myo7a$^{fl/fl}$Myo15-cre$^{+/-}$* mice
(***A,B***) Cochlear whole mount preparations from control *Myo7a$^{fl/fl}$* (***A***) and *Myo7a$^{fl/fl}$Myo15-cre$^{+/-}$* mice (***B***) immunostained for MYO7A (green). DAPI (blue) is the nuclear marker. Note that MYO7A was no longer detected in the OHCs at P20.

SI Appendix **Figure S6**

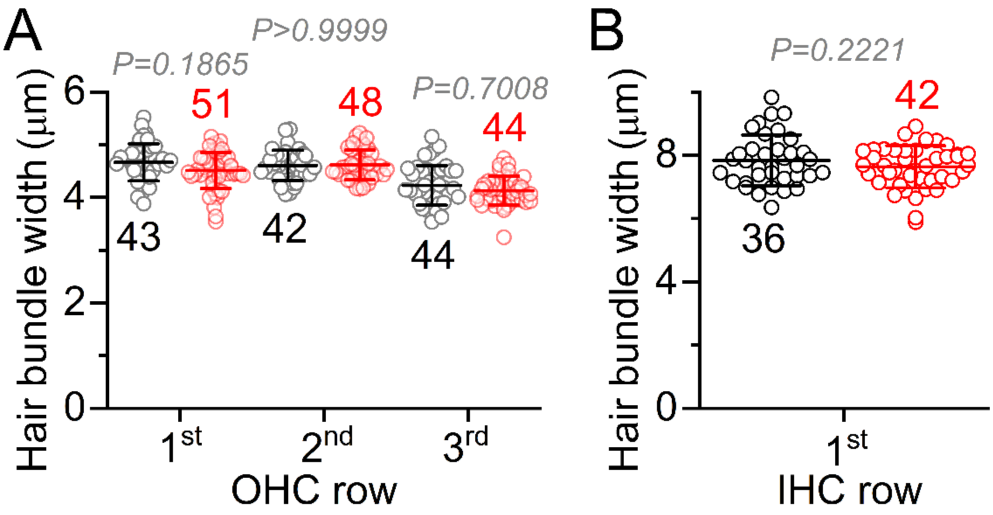

Fig. S6: Width of the hair bundles of both adult OHCs and IHCs in adult *Myo7a* knockout mice (***A,B***) Width of the hair bundles of the three rows of OHCs (***A***) and one row of IHCs (***B***) from adult mice of both genotypes. Numbers above or below the data points indicate the hair cells investigated from the same mice listed for **Fig. 3*I,J*** (OHCs) and **Fig. 4*I-K*** (IHCs). Statistical values in panel (***A***) were from the Tukey post-test, one-way ANOVA; in panel (***B***) from a *t*-test.

SI Appendix Figure S7

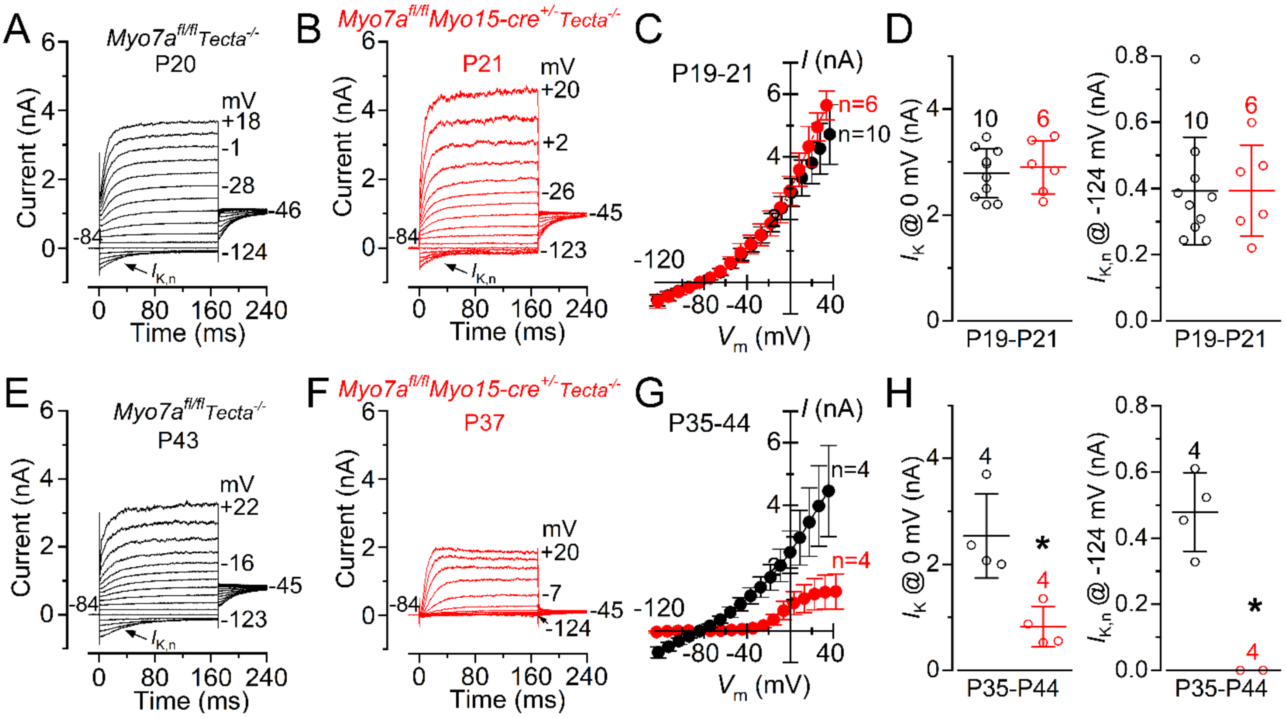

Fig. S7: The basolateral membrane properties of adult OHCs are lost in *Myo7a* knockout mice (***A,B***) Current responses from OHCs of control P20 $Myo7a^{fl/fl}Tecta^{-/-}$ and P21 $Myo7a^{fl/fl}Myo15\text{-}cre^{+/-}Tecta^{-/-}$ mice. Current recordings were elicited by using depolarizing and hyperpolarizing voltage steps (10 mV increments) from the holding potential of –84 mV to the various test potentials shown by some of the traces. Mature OHCs expressed a large outward K^+ current carried by KCNQ4 channels called $I_{K,n}$ (**18,19**). $I_{K,n}$ is best identified by the de-activating tail currents at negative membrane potentials, and it was present in the OHCs of both genotypes. (***C***), Peak total current plotted as a function of membrane potential from $Myo7a^{fl/fl}Tecta^{-/-}$ mice (10 OHCs from 7 mice, P19-P21) and $Myo7a^{fl/fl}Myo15\text{-}cre^{+/-}Tecta^{-/-}$ mice (6 OHCs from 4 mice, P19-P21). (***D***), Average size of the peak total outward K^+ current measured at 0 mV (left: I_K) and the isolated $I_{K,n}$, which was measured as the difference between the peak and steady-state of the deactivating inward current at -124 mV, were indistinguishable between the two genotypes (I_K: $P = 0.6563$; $I_{K,n}$: $P = 0.9836$, *t*-test). Overall, these data show that at around P20, which is a time when both DPOAE thresholds and the MET current in OHCs were normal in the mice lacking MYO7A (**Figs 1,3**), the size of $I_{K,n}$ was normal in both $Myo7a^{fl/fl}Tecta^{-/-}$ and littermate $Myo7a^{fl/fl}Myo15\text{-}cre^{+/-}Tecta^{-/-}$ mice.

(***E,F***) Current responses from OHCs of P43 $Myo7a^{fl/fl}Tecta^{-/-}$ and P37 $Myo7a^{fl/fl}Myo15\text{-}cre^{+/-}Tecta^{-/-}$ mice. Current recordings were obtained as described in panels ***A,B***. (***G***), Peak total current plotted as a function of membrane potential from $Myo7a^{fl/fl}Tecta^{-/-}$ mice (4 OHCs from 3 mice, P35-P43) and $Myo7a^{fl/fl}Myo15\text{-}cre^{+/-}Tecta^{-/-}$ mice (4 OHCs from 3 mice, P35-P44). (***H***), Average size of the peak total outward K^+ current measured at 0 mV (left: I_K) and the isolated $I_{K,n}$, which were significantly reduced in the OHCs from $Myo7a^{fl/fl}Myo15\text{-}cre^{+/-}Tecta^{-/-}$ mice compared to those in $Myo7a^{fl/fl}Tecta^{-/-}$ mice (I_K: $P = 0.0082$; $I_{K,n}$: $P < 0.0001$, *t*-test). Overall, these data show that by P35, about 10 days after the MET current became strongly downregulated (**Fig. 3**), the OHCs had completely lost $I_{K,n}$, leaving a small delayed rectifier outward K^+ current that is reminiscent of pre-hearing OHCs (**18**).

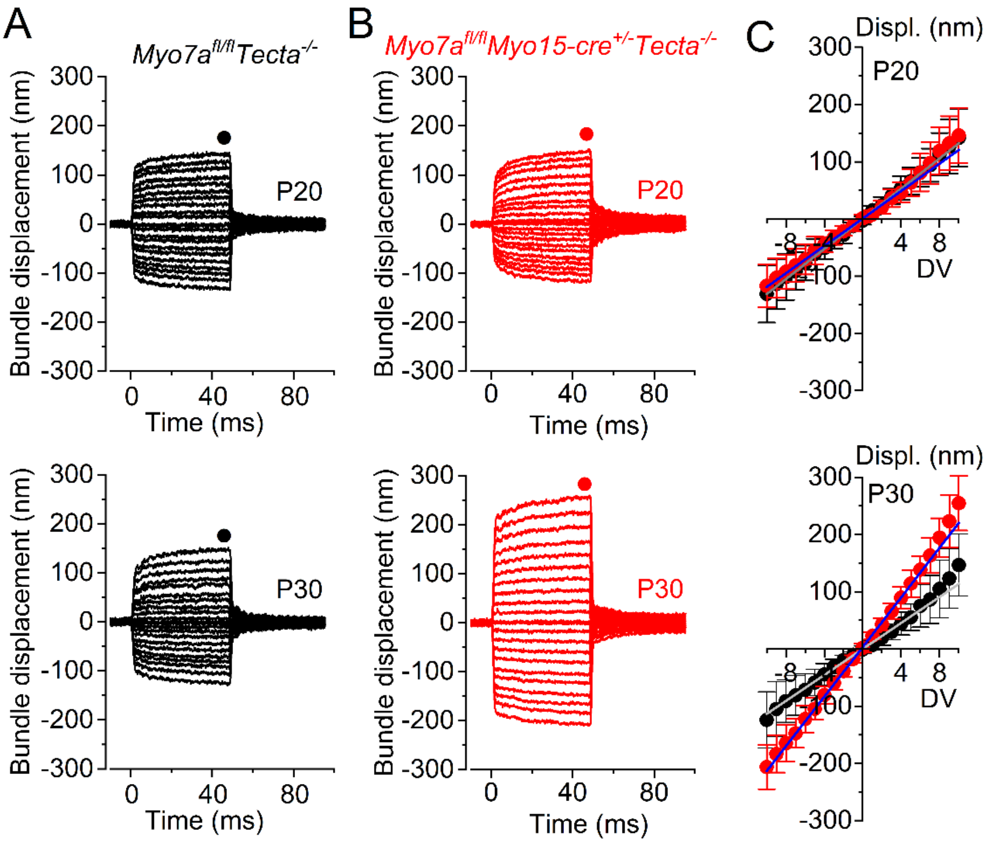

Fig. S8: Adult OHCs from *Myo7a* knockout mice have a larger hair bundle displacement. (***A, B***) Average hair bundle displacement of OHCs from P20 (top panels) and P30 (bottom panels) control (***A***) and *Myo7a*$^{fl/fl}$*Myo15-cre*$^{+/-}$*Tecta*$^{-/-}$ mice (***B***) elicited by applying incremental 1V force-step stimuli up to ±10V. (***C***) Average steady-state bundle displacement at P20 (left panel) and P30 (right panel) plotted against the driver voltage to the fluid jet from panels ***A,B***. The slope of the linear fit (bundle displacement *vs* driver voltage) from individual hair bundle recordings expressed in V/nm was used to measure the apparent overall steady-state bundle stiffness for both genotypes showed in the main **Fig. 5*F***.

***SI Appendix* Figure S9**

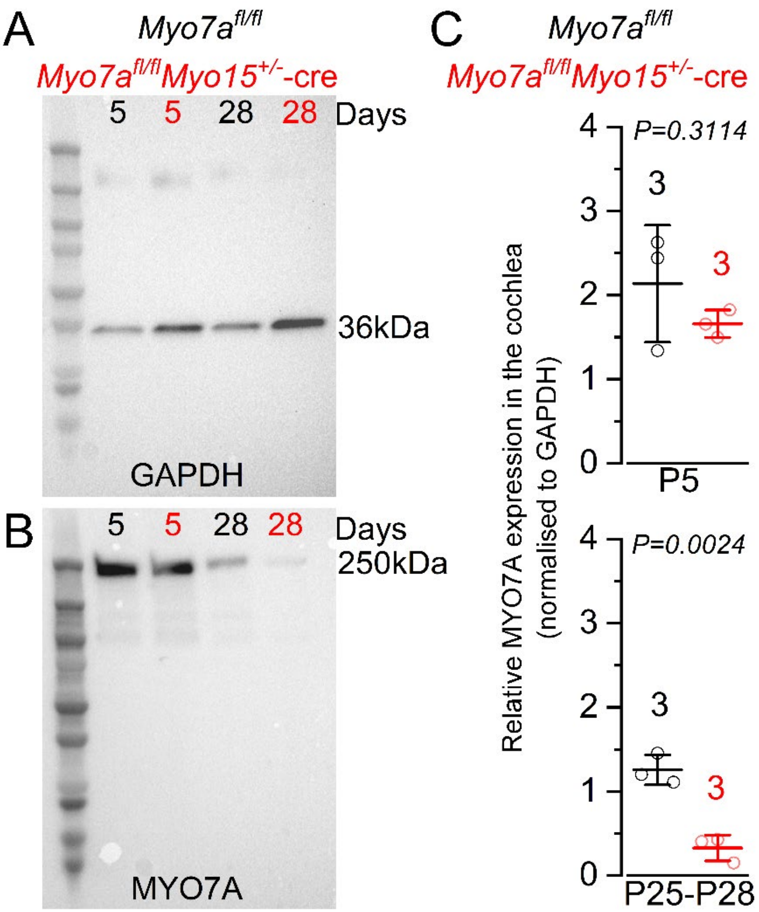

Fig. S9: Residual MYO7A is present in the cochlea of *Myo7a*-deficient mice
Western blot showing MYO7A expression at P5 and P25-P28 in the cochlea of control (*Myo7a*$^{fl/fl}$) and *Myo7a*$^{fl/fl}$*Myo15-cre*$^{+/-}$ mice, with GAPDH used as a loading control for normalization. Full gel run from one of the western blots showing both GAPDH (***A***) and MYO7A (***B***). Data were collected from 3 separate experiments, with each sample containing the cochlea of 2 mice for each genotype. Data are reported as mean ± SD. Statistical analysis was performed using *t*-tests.

SI Appendix Figure S10

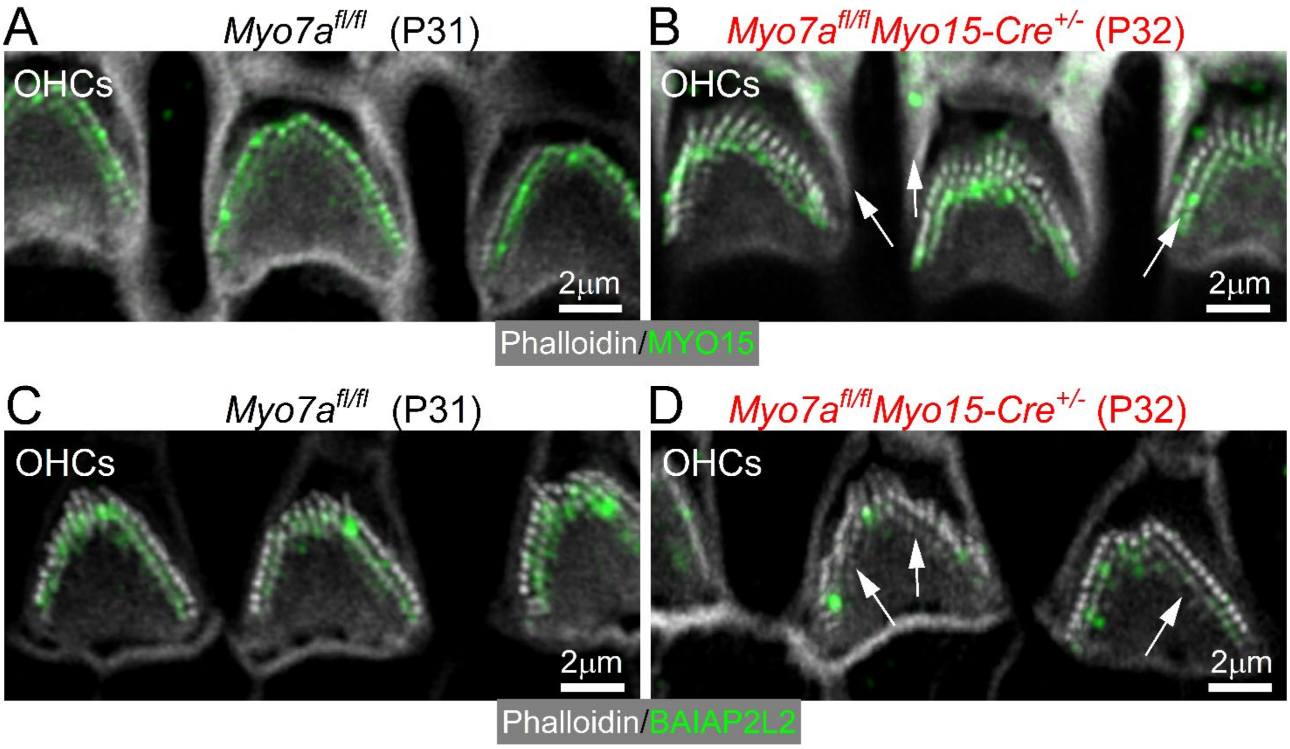

Fig. S10: Localization of the bundle proteins MYO15 and BAIAP2L2 in the OHCs of adult *Myo7a*-deficient mice

(***A,B***) Confocal images of the hair bundles from apical-coil OHCs of P31 control *Myo7a*$^{fl/fl}$ (***A***) and littermate *Myo7a*$^{fl/fl}$*Myo15-cre*$^{+/-}$ mice (***B***) immunostained with an antibody against MYO15 (green). Note the normal distribution pattern of MYO15 at the tip of the stereocilia in both genotypes. (***C, D***) Confocal images of the hair bundles from apical-coil OHCs of P31 *Myo7a*$^{fl/fl}$ (***C***) and littermates *Myo7a*$^{fl/fl}$*Myo15-cre*$^{+/-}$ mice (***D***) immunostained with an antibody against BAIAP2L2. Note the characteristic localization of BAIAP2L2 puncta at the top of the shorter rows of stereocilia of the control *Myo7a*$^{fl/fl}$ mice (***C***). In the knockout mice, however, the staining was largely reduced (***D***, arrows). The stereocilia actin core was labelled with phalloidin (white). All experiments were performed in 3 mice per genotype.

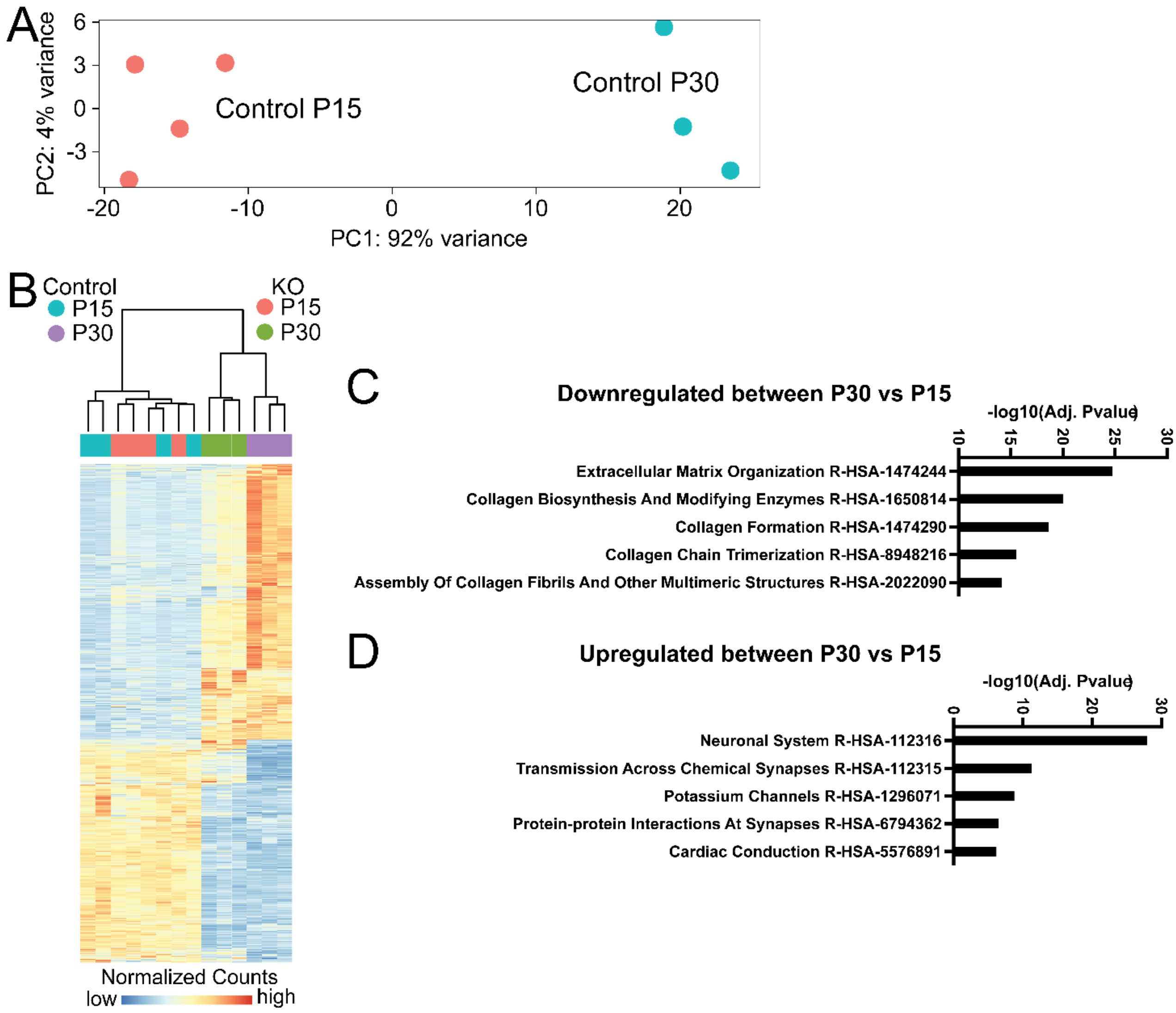

Figure S11: Differentially expressed genes from P15 to P30 cochlear tissue in control *Myo7a*$^{fl/fl}$ mice

(***A***) Principal Component Analysis (PCA) of the top 2000 most variable genes in each cDNA library at P15 and P30 in control *Myo7a*$^{fl/fl}$ mice. PC1, which explains 91% of the variance, divides the two ages. (***B***) Heatmap of the differentially expressed genes between P15 and P30 *Myo7a*$^{fl/fl}$ mice, with the *Myo7a*$^{fl/fl}$*Myo15-cre*$^{+/-}$ mice plotted as a reference. Only a subset of the genes regulated during cochlear maturation are altered in knockout mice. (***C,D***) Pathway analysis of the downregulated (***C***) and upregulated (***D***) genes between P15 and P30. A full list of terms is available in ***SI Appendix* Table 5**).

***SI Appendix* Figure S12**

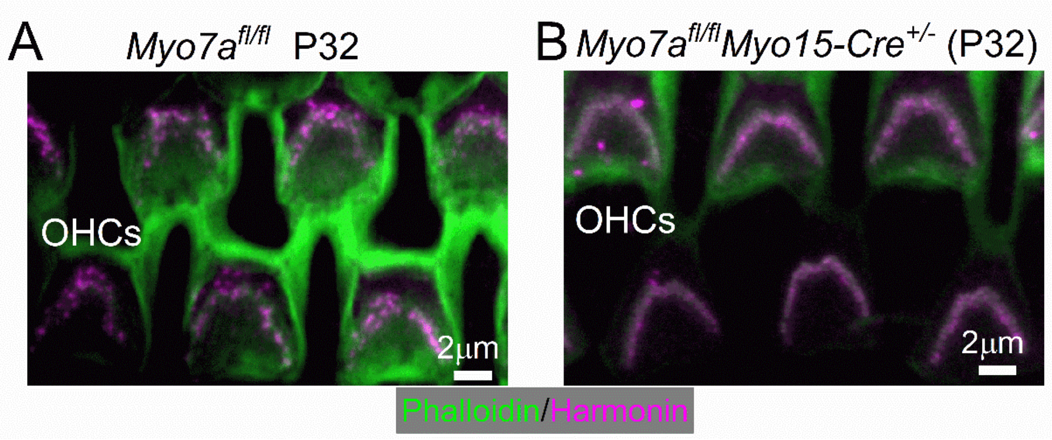

Fig. S12: Localization of the bundle protein Harmonin in the OHCs from adult *Myo7a*-deficient mice

(***A*,*B***) Confocal images of the hair bundles from apical-coil OHCs of P32 control *Myo7a*$^{fl/fl}$ (***A***) and littermate *Myo7a*$^{fl/fl}$*Myo15-cre*$^{+/-}$ mice (***B***) immunostained with an antibody against Harmonin (magenta). Note the normal distribution pattern of harmonin at the tip of the stereocilia in both genotypes. The stereocilia actin core was labelled with phalloidin (green). Experiments were performed in 3 mice per genotype.

SI References

1. P.K. Legan, *et al.,* A targeted deletion in alpha-tectorin reveals that the tectorial membrane is required for the gain and timing of cochlear feedback. *Neuron* **28**, 273-285 (2000).
2. A.J. Carlton, *et al.,* Loss of Baiap2l2 destabilizes the transducing stereocilia of cochlear hair cells and leads to deafness. *J. Physiol.* **599**, 1173-1198 (2021).
3. N.J. Ingham, S. Pearson, K.P. Steel, Using the auditory brainstem response (ABR) to determine sensitivity of hearing in mutant mice. *Curr. Protoc. Mouse Biol.* **1**, 279–287 (2011).
4. R.H., Holme, K.P. Steel, Progressive hearing loss and increased susceptibility to noise-induced hearing loss in mice carrying a Cdh23 but not a Myo7a mutation. *J. Assoc. Res. Otolaryngol.* **5**, 66-79 (2004).
5. A.J. Carlton, *et al.,* A critical period of prehearing spontaneous Ca2+ spiking is required for hair-bundle maintenance in inner hair cells. *EMBO J.* **42**, e112118 (2023).
6. J.Y. Jeng, *et al.,* Age-related changes in the biophysical and morphological characteristics of mouse cochlear outer hair cells. *J. Physiol.* **598**, 3891-3910 (2020).
7. L.F. Corns, S.L. Johnson, C.J. Kros, W. Marcotti, Calcium entry into stereocilia drives adaptation of the mechanoelectrical transducer current of mammalian cochlear hair cells. *Proc. Natl. Acad. Sci. USA* **111**, 14918-14923 (2014).
8. W. Marcotti, *et al.,* The acquisition of mechano-electrical transducer current adaptation in auditory hair cells requires myosin VI. *J. Physiol.* **594**, 3667-3681 (2016).
9. L.F. Corns, *et al.,* Mechanotransduction is required for establishing and maintaining mature inner hair cells and regulating efferent innervation. *Nat. Comm.* **9**, 4015 (2018).
10. G.S.G. Géléoc, G.W.T. Lennan, G.P. Richardson, C.J. Kros, A quantitative comparison of mechanoelectrical transduction in vestibular and auditory hair cells of neonatal mice. *Proc. Biol. Sci.* **264**, 611–621 (1997).
11. D.N. Furness, C.M. Hackney, High-resolution scanning-electron microscopy of stereocilia using the osmium-thiocarbohydrazide coating technique. *Hear. Res.* **21**, 243–249 (1986).
12. L.A. Dunbar *et al.,* Clarin-2 is essential for hearing by maintaining stereocilia integrity and function. *EMBO. Mol. Med.* **11**, e10288 (2019).
13. P.A. Ewels, *et al.,* The nf-core framework for community-curated bioinformatics pipelines. *Nat. Biotechnol.* **38**, 276-278 (2020).
14. R. Patro, G. Duggal, M.I. Love, R.A. Irizarry, C. Kingsford, Salmon provides fast and bias-aware quantification of transcript expression. *Nat. Methods* **14**, 417-419 (2017).
15. M.I. Love, W. Huber, S. Anders, Moderated estimation of fold change and dispersion for RNA-seq data with DESeq2. *Genome Biol.* **15**, 550 (2014).
16. Y. Zhou, *et al.,* Metascape provides a biologist-oriented resource for the analysis of systems-level datasets. *Nat. Comm.* **10**, 1523 (2019).
17. M. Gillespie, *et al.,* The reactome pathway knowledgebase 2022. *Nucleic Acids Res.* **50**, D687-D692 (2022).
18. W. Marcotti, C.J. Kros, Developmental expression of the potassium current IK,n contributes to maturation of mouse outer hair cells. *J. Physiol.* **520**, 653-660 (1999).
19. C. Kubisch, *et al.,* KCNQ4, a novel potassium channel expressed in sensory outer hair cells, is mutated in dominant deafness. *Cell* **96**, 437–446 (1999).
